# French recommendations for the management of systemic sclerosis

**DOI:** 10.1186/s13023-021-01844-y

**Published:** 2021-07-26

**Authors:** Eric Hachulla, Christian Agard, Yannick Allanore, Jerome Avouac, Brigitte Bader-Meunier, Alexandre Belot, Alice Berezne, Anne-Sophie Bouthors, Geraldine Condette-Wojtasik, Joël Constans, Pascal De Groote, Elisabeth Diot, Florence Dumas, Patrick Jego, Francisca Joly, David Launay, Veronique Le Guern, Janine-Sophie Le Quintrec, Geraldine Lescaille, Christophe Meune, Bruno Moulin, Christelle Nguyen, Nadine Omeish, Frederic Pene, Marie-Aleth Richard, Juliette Rochefort, Alexandra Roren, Olivier Sitbon, Vincent Sobanski, Marie-Elise Truchetet, Luc Mouthon, Collaborators, Marc Bayen, Emmanuel Bergot, Sabine Berthier, Julia Bosco, Yoram Bouhnik, Benjamin Chaigne, Vincent Cottin, Bruno Crestani, Christophe Deligny, Vianney Descroix, Dominique Farge, Dominique Godard, Brigitte Granel, Philippe Guilpain, Bernard Imbert, Alain Le Quellec, Christophe Lega, Catherine Lok, Hélène Maillard, Thierry Martin, Grégory Pugnet, Viviane Queyrel, Loïc Raffray, Frédéric Rilliard, Mélanie Romier, Laurence Schuller, Amélie Servettaz

**Affiliations:** 1 Service de Médecine Interne et Immunologie Clinique, Centre de Référence Des Maladies Autoimmunes Systémiques Rares du Nord et Nord-Ouest de France (CeRAINO), Univ. Lille, Inserm, CHU Lille, U1286 - INFINITE - Institute for Translational Research in Inflammation, 59000 Lille, France; 2 Internal Medicine, Nantes University Hospital, University of Nantes, Nantes, France; 3 Rheumatology Department, Hôpital Cochin, AP-HP, Université de Paris, Paris, France; 4 Department of Pediatric Immunology and Rheumatology; Hospital Necker, APHP, Paris, France; 5 Pediatric Nephrology, Rheumatology, Dermatology, HFME, Hospices Civils de Lyon, Bron, France; 6 Department of Internal Medicine, CHR Annecy-Genevois, Annecy, France; 7 Anaesthesia Intensive Care Unit, Jeanne de Flandre Women Hospital, Academic Hospital, ULR 7365 - GRITA - Groupe de Recherche Sur Les Formes Injectables Et Les Technologies Associées, University Lille, Lille, France; 8 Vascular Medicine Department, Bordeaux University Hospital Centre, Saint André Hospital, FCRIN INI-CRCT (Cardiovascular and Renal Clinical Trialists) PeripherAL Artery DIsease Network (PALADIN), Bordeaux, France; 9 Cardiology Department, Lung-Heart Institute, CHU de Lille, 59000 Lille, France; 10 Service de Médecine Interne, CHU Tours, Tours, France; 11 Emergency Department, Cochin Hospital, Paris University, Paris, France; 12 Internal Medicine and Clinical Immunology Unit, CHU Rennes, Rennes, France; 13 Department of Gastroenterology, IBD and Nutrition Support, Beaujon Hospital, INSERM UMRS-1149, Assistance Publique-Hôpitaux de Paris, University of Paris, Clichy, France; 14 Service de Médecine Interne, Centre de Référence Maladies Autoimmunes Systémiques Rares D’Ile de France, Hôpital Cochin, Assistance Publique-Hôpitaux de Paris (AP-HP), APHP-CUP, Hôpital Cochin, Université de Paris, 75014 Paris, France; 15 Centre d’Immunologie et Maladies Infectieuses (CIMI-Paris), Department of Odontology, Paris Diderot/Paris 07, Sorbonne Paris Cité, AP-HP, Groupe Hospitalier Pitié-Salpêtrière, Paris, France; 16 Cardiology Department, Hôpital Avicenne, AP-HP, Université de Paris, Paris, France; 17 Department of Nephrology and Kidney Transplantation, Nouvel Hôpital Civil, University Hospitals of Strasbourg, Strasbourg, France; 18 Physical Medicine and Rehabilitation Department, Hôpital Cochin, AP-HP, Université de Paris, Paris, France; 19 Oral and Dental Medicine, Hôpital Pitié-Salpêtrière, APHP, Université de Paris, Paris, France; 20 Medical Intensive Care Unit, Hôpital Cochin, AP-HP. Centre & Université de Paris, Paris, France; 21 Department of Dermatology, Timone Hospital, University Hospital of Marseille, Marseille, France; 22 AP-HP Cochin Hospital, Université Paris Descartes Sorbonne Paris Cité, INSERM U1153, Paris, France; 23 Assistance Publique-Hôpitaux de Paris, Service de Pneumologie, Hôpital Bicêtre, Laboratoire d’Excellence en Recherche Sur le Médicament et Innovation Thérapeutique, Université Paris-Sud, Le Kremlin-Bicêtre, France; 24 Rheumatology Department, Hôpital Pellegrin, CHU de Bordeaux, Bordeaux, France

**Keywords:** Systemic sclerosis, Recommendations, Treatment

## Abstract

Systemic sclerosis (SSc) is a generalized disease of the connective tissue, arterioles, and microvessels, characterized by the appearance of fibrosis and vascular obliteration. There are two main phenotypical forms of SSc: a diffuse cutaneous form that extends towards the proximal region of the limbs and/or torso, and a limited cutaneous form where the cutaneous sclerosis only affects the extremities of the limbs (without passing beyond the elbows and knees). There also exists in less than 10% of cases forms that never involve the skin. This is called SSc sine scleroderma. The prognosis depends essentially on the occurrence of visceral damage and more particularly interstitial lung disease (which is sometimes severe), pulmonary arterial hypertension, or primary cardiac damage, which represent the three commonest causes of mortality in SSc. Another type of involvement with poor prognosis, scleroderma renal crisis, is rare (less than 5% of cases). Cutaneous extension is also an important parameter, with the diffuse cutaneous forms having less favorable prognosis.

## Summary

### Initial assessment

**Fig. 1:**
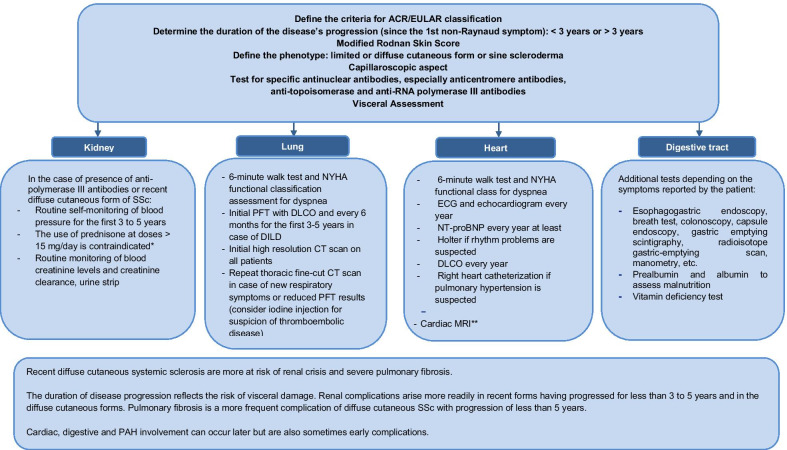
Recommendations for initial evaluation and follow-up of patients with systemic sclerosis. *ACR* American College of Rheumatology, *EULAR* European League Against Rheumatism, *RNA* ribonucleic acid, *NYHA* New York Heart Association, *PFT* pulmonary function tests, *DLCO* carbon monoxide diffusing capacity, *ILD* diffuse infiltrative lung disease, *ECG* electrocardiogram, *NT-proBNP* N-terminal pro b-type natriuretic peptide, *MRI* magnetic resonance imaging, *PAH* pulmonary arterial hypertension. *Risk of scleroderma renal crisis, **if cardiac damage is in doubt

The severity of visceral damage justifies systematic and repeated assessment by directed interview and clinical and additional examinations, even in the absence of suggestive symptoms, as early treatment is a determining factor for patient survival (Fig. 1).

### Therapeutic management

DMARDs can be proposed based on the type of clinical presentation and possible visceral damage.

To date, no antifibrotic or immunosuppressant DMARD monotherapy has been able to achieve an improvement in survival in a prospective randomized study. Use of cyclophosphamide at very high doses during intensive conditioning procedures for myelosuppressive or myeloablative purposes (depending on the type of conditioning) with or without antilymphocytic serum followed by autologous hematopoietic stem cell (HSC) transplantation has been proven efficacious in terms of event-free and overall survival in three randomized trials (ASSIT, ASTIS, and SCOTT). These therapeutic procedures are reserved for severe, rapidly progressive forms, after rigorous selection of patients, and must be carried out in special centers accredited for these procedures.

Nonpharmacological therapies (such as functional rehabilitation) are in all cases recommended to combat disability.

Therapeutic patient education (TPE) must ensure that the SSc patient and their circle of family and friends have a good understanding of the disease. TPE complements and is an integral part of the patient’s treatment and care. It can contribute to relieving symptoms and preventing complications. TPE contributes to improving the patient’s health and quality of life, along with that of their circle of family and friends. TPE enables patients to acquire and maintain the skills they need to better manage their lives with a chronic illness (Chapter 5.3).

The information should include:

- The different types of SSc, their symptoms, specifying warning signs: appearance of dyspnea, HBP, kidney failure, aggravation of Raynaud's phenomenon and the appearance of a digital ulcer, onset of anemia, dizziness, syncopes, severe digestive disorders such as an occlusive condition, profuse diarrhea, dysphagia, etc., which should lead the patient to seek immediate medical care. Any change or aggravation of symptoms should lead the patient to seek medical care;
- Available and prescribed treatments, and possible adverse effects of those received by the patient;
- The planning of relevant additional examinations to detect possible complications and the expected results.

#### Patient associations

Healthcare professionals and patients must be informed of the existence of patient associations, institutional websites, and Orphanet, by reference and competent centers, and healthcare professionals who are experts in the care of scleroderma patients.

#### Treatments

Treatments should target especially visceral damage due to SSc. If antifibrotic treatments are a route in the future, therapeutic choices at the individual level must be conditioned by complete initial visceral assessment, evaluation of the activity and prognosis of SSc based on regular follow-up, and assessment of comorbidities.

### Follow-up

The frequency of medical consultations should be based on the initial severity, activity and length of the disease, type of visceral damage, and/or appearance of intercurrent events. Physical examination is needed with every modification of treatment (Fig. 1).

Generally speaking, a physical examination is needed:

- Quarterly for the first 3 years of progression (from the first non-Raynaud’s phenomenon symptom) of the diffuse cutaneous forms, and less frequently thereafter;
- Every 6 months in the case of the limited cutaneous form and in the absence of visceral damage;
- More frequently in case of modification or aggravation of symptoms.

An assessment of complications and visceral damage is performed at least annually, and sometimes more frequently, particularly in case of recently diagnosed diffuse cutaneous forms.

Particular circumstances include:

- Situations of urgency (Appendix 7);
- Recommendations before anesthesia (Appendix 6).

## Objectives

The objective of this national diagnostic and care protocol (NDCP) is to explain for healthcare professionals the best treatment and course of care for a patient with long-term illness under the title of ALD 21: “periarteritis nodosa, acute disseminated lupus erythematosus, SSc.”

This NDCP solely concerns patients afflicted by SSc and is limited to the form indicated in the text of current ALD regulations. It was decided that this NDCP would not include localized sclerodermas, also called morphea, which are sclerotic attacks of the skin, most often limited to it, but which can sometimes extend to the underlying muscle, bone, and nerve tissues, without systemic manifestations.

It is a practical tool to which the attending physician, in consultation with other medical specialists, can refer for the management of the disease in question, in particular when establishing the treatment protocol^1^ together with the consulting physician and the patient. The treatment protocol is now established directly on the Amelipro website, where it is necessary only to provide information on the disease. This NDCP will therefore be useful to determine the elements of care accepted within the framework of ALD 21.

This NDCP cannot, however, consider all specific cases, comorbidities, therapeutic particularities, hospital care protocols, etc. It cannot claim to include all possible management procedures or replace the individual responsibility of the doctor toward his/her patient. Nonetheless, this protocol does reflect the essential structure of treatment of the patient with SSc, and will be updated as new information is validated.

## Systemic sclerosis

### Definition

SSc is a rare disease in which visceral manifestations may occur, in particular peripheral vascular, digestive, cardiopulmonary, and renal.

It is characterized by anomalies of the microcirculation (more rarely of the macrocirculation, although this aspect is still being debated) and by cutaneous and/or visceral fibrotic lesions. Cutaneous sclerosis lesions can be modest, distal (on the fingers especially), and around the mouth, or extend above the elbows and knees, or more rarely reach the torso and abdomen. There is also a rare form without cutaneous involvement, called “sine scleroderma.”

### Epidemiology

SSc predominantly affects women (three to eight women for each man). It occurs most frequently between the ages of 45 and 64 years. The prevalence of SSc is still poorly understood. In France, the prevalence of SSc was 158 cases per million adult inhabitants in a study conducted in 2004 in the Department of Seine Saint-Denis, 132 cases per million adult inhabitants in a study conducted in Loraine published in 2013, and 228 cases per million adult inhabitants in a study conducted in Alsace and published in 2016, allowing, by extrapolation, as estimation of the number of adult SSc patients in France at 6000–9000. SSc is extremely rare in children, representing less than 5% of all SSc cases. An English study evaluated its incidence at 0.27 per million children, with a female-to-male sex ratio of 3.6.

### Physiopathology

The physiopathology of SSc is complex and still poorly understood, being associated with endothelial cell, fibroblastic, and immune system dysfunction.

Fibroblastic dysfunction is characterized by uncontrolled activation, leading to excessive protein synthesis in extracellular matrix. Endothelial cells synthesize excess endothelin 1, a powerful vasoconstrictor. Antinuclear antibodies (Ab) are detectable in the serum of the majority of patients, targeting nuclear proteins. Other Abs recognize the endothelial cells and/or fibroblasts. Oxidative stress seems to play a major role in the pathogenesis of SSc. All of these anomalies could be associated with genetic predisposition.

SSc is sometimes promoted by exposure to certain environmental factors such as silica and solvents, often in a professional context. A declaration of occupational illness (French Table 25 of the occupational diseases) can be made for silica, whether it relates to exposure or to confirmed silicosis. Exposure to other substances (solvents, welding fumes, etc.) are considered occupational illnesses. Claims must then be filed with the regional recognition committee.

### Classification of systemic sclerosis

New classification criteria for SSc were established by EULAR and ACR (Table 1), providing enhanced sensitivity and specificity for the classification of patients compared with previous criteria. The score obtained using these criteria should not be understood as a severity score. Nevertheless, any clinical suspicion of SSc should lead to an antinuclear antibody test and capillaroscopy. Cardiopulmonary assessment must be conducted next.

**Table 1:** ACR–EULAR classification criteria for systemic sclerosis

Domain	Criteria*	Score^#^
Cutaneous thickening (only consider the highest score)	Cutaneous thickening of the fingers extending beyond the MCP joints	9
	Swollen fingers	2
	Symptoms of the fingers not extending beyond the MCP joints	4
Pulp lesions (only consider the highest score)	Digital pulp ulcers	2
	Depressed scars	3
Telangiectasias		2
Capillaroscopic anomalies		2
Pulmonary damage	PAH and/or pulmonary fibrosis	2
Raynaud’s phenomenon		3
SSc-specific antibodies	Anti-topoisomerase I	3
	Anticentromere antibodies	
	Anti-RNA polymerase III	

*RNA* ribonucleic acid, *MCP* metacarpophalangeal, *SSc* systemic sclerosis

^*^This criterion can be included if appearing at any time during the clinical history

^#^The weight of each item present must be summed to obtain the total score. A score of 9 or more permits classifying patients as having SSc

According to LeRoy et al. [58], systemic sclerodermas can be classified into three main phenotypes:

- Diffuse cutaneous SSc, when the cutaneous sclerosis extends above the elbows and/or knees
- Limited cutaneous SSc, when the cutaneous sclerosis does not extend above the elbows and knees
- SSc sine scleroderma, in the absence of cutaneous sclerosis

### Progress and prognosis

The progress and prognosis of the disease can vary widely and depend on the cutaneous form of the SSc as well as on the presence of visceral damage. Diffuse cutaneous SSc is defined by rapidly progressing skin lesions with maximum extension in 1–5 years after the appearance of the first clinical sign other than Raynaud’s phenomenon. In these forms, visceral manifestations appear especially in the first 3–5 years in the form of muscular and digestive symptoms, renal crisis, diffuse infiltrative lung disease, and/or cardiac symptoms. Beyond this period, other visceral manifestations can appear, especially pulmonary arterial hypertension (PAH). Limited cutaneous SSc is less frequently accompanied by visceral manifestations but can be complicated by PAH along with severe digestive manifestations during its progress. Also, mortality is higher in the diffuse than limited cutaneous form of the disease. Consequently, management of patients having the diffuse form differs from that of patients with the limited form.

Studies show that SSc is responsible for a significant drop in survival, with a 10-year survival rate of around 60% for the diffuse cutaneous form and 80% for the limited form. Life expectancy depends essentially on the presence of visceral damage, and more particularly on the presence of pulmonary involvement [severe interstitial lung disease (ILD), 14% of cases], PAH (5–10% of cases), or specific cardiac involvement (with 3% of patients having a change in left ventricular ejection fraction), with these three representing the main causes of death in SSc patients.

The severity of the visceral damage justifies systematic and repeated testing by directed interview and clinical and additional examinations, and for some of them, such as PAH and ILD, even in the absence of symptoms. In fact, their early treatment is a determining factor for patient survival and constitutes an essential objective of SSc care. All this explains why monitoring at an expert center must occur at least annually and adapted according to the type of complications, with closer monitoring in the event of severe or progressive visceral damage and in recent forms of less than 3–5 years, especially for diffuse cutaneous SSc.

### Treatments

Disease-modifying drugs can be proposed based on the type of clinical presentation and possible visceral damage.

Particular care must be given to symptomatic drug treatments. Moreover, nonpharmacological therapies such as functional rehabilitation can reduce disability with regard to SSc, but their efficacy depends on patient adherence to the treatment.

**Fig. 2:**
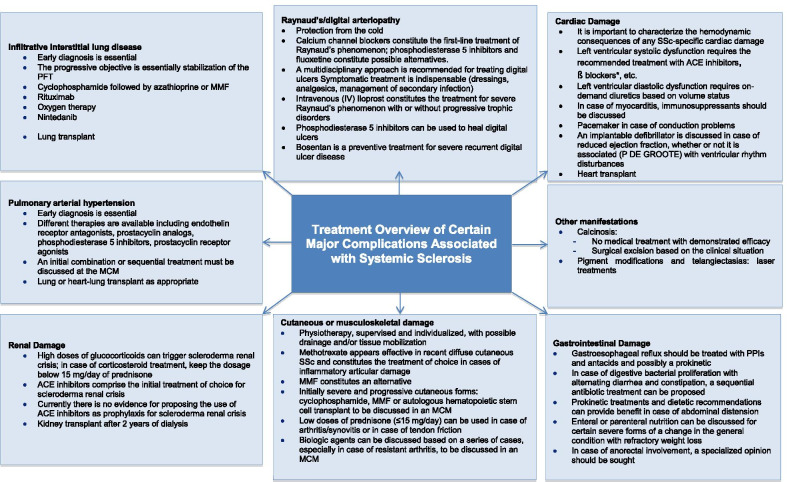
Possible therapeutic approaches to the main visceral complications of systemic sclerosis. *Cardioselective blockers to be discussed according to PAH and Raynaud’s/digital ulcers if no therapeutic alternative. *PFT* pulmonary function tests, *LV* left ventricle, *IV* intravenous, *MCM* multidisciplinary coordination meeting, *SSc* systemic scleroderma, *ACE* angiotensin-converting enzyme, *MMF* mycophenolate mofetil

Figure 2 summarizes the main therapeutic principles proposed for SSc on the basis of expert opinion and the recommendations of EULAR 2017.

## Method

This NDCP has been drafted based on a critical analysis of the international literature, according to the “Method of Drafting the National Diagnostic and Care Protocol for Rare Diseases” published by the HAS (2012).

The contents of this NDCP was discussed and validated by a multidisciplinary working group.

## Initial assessment of systemic sclerosis

The clinical presentation of SSc is very heterogeneous. Initial clinical examinations must look for the elements necessary for the diagnosis of diffuse or limited cutaneous SSc, and screen for visceral complications of the disease. In the initial assessment stage, most patients satisfy the EULAR–ACR 2013 classification criteria (Table 1).

### Main objectives

- Confirm the diagnosis of SSc
- Look for factors related to occupational exposure (silica, solvents) and associated/aggravating factors of Raynaud’s phenomenon (vibration, hammering, outlet syndrome, etc.)
- Specify the type of initial cutaneous involvement: diffuse or limited
- Look for visceral damage and assess its severity
- Determine prognosis based on the visceral damage
- Screen for autoimmune pathologies most frequently associated with SSc (Sjögren’s syndrome, Hashimoto’s disease, primary biliary cholangitis, etc.)
- Formulate the therapeutic indications

### Professionals involved

The initial management of the SSc patient is provided by:

- A physician specializing in SSc from an expert center: internist, dermatologist, vascular specialist, rheumatologist, or pediatrician
- Other medical specialists who may potentially be involved: pneumologists, cardiologists, nephrologists, hepatogastroenterologists, physiatrists, psychiatrists, stomatologists (oral surgeons), orthopedic surgeons, hand surgeons, nutritionists, and other health professionals (occupational therapists, physical therapists, psychologists, dieticians, etc.)
- Reference centers, competence centers, or expert centers and their networks of collaborators
- The general practitioner
- Any other specialist whose opinion is necessary based on the clinical profile

### Clinical examination

The *patient interview* looks for possible occupational exposure to silica or solvents, for the purposes of specifying the risk factors and conducting a professional investigation. It will also be necessary to look for factors that could aggravate vasculopathy (hammering, vibrations) as well as exposure to tobacco and recreational drugs (cannabis, etc.).

It determines the impact on daily activity and especially on professional activity.

#### The physical examination seeks objective elements necessary for the diagnosis

The diagnosis should be derived from the association of several criteria defined in the ACR–EULAR classification (Table 1). Other symptoms are possible, such as gastroesophageal reflux, subcutaneous calcifications, arthritides/synovitises, or renal crisis.

- Raynaud’s phenomenon (> 95% of cases)

- It is frequently associated with trophic problems such as pulp scarring and digital ulcers.
- It sometimes occurs alone for several years before other signs of SSc appear. It can, however, be concurrent with cutaneous or visceral involvement.

- Cutaneous and mucosal signs
  These are sometimes absent (as in scleroderma sine scleroderma). With SSc, the skin modification(s) often confirm(s) the diagnosis. Cutaneous sclerosis is sometimes absent or not yet present. It can progress in three phases:

- *Edematous phase* inconsistent, observed especially in the diffuse form, characterized by swelling of the fingers and hands, sometimes of the limbs, which are infiltrated.
- *Indurative phase* cutaneous thickening. The skin cannot be creased and may adhere to deep tissues. Facial sclerosis is responsible for the disappearance of wrinkles on the forehead, a thin, pinched nose, a reduction in the opening of the mouth measured by the distance between the dental arches (if < 40 mm).
- *Atrophic phase* Atrophy and then disappearance of the hypodermis, resulting in thin skin, thinned appearance of the lips, and exaggeration of peribuccal radiating creases. Sometimes, the skin can return to normal.

The technique most widely used to assess the extent of cutaneous sclerosis is calculation of the modified Rodnan skin score (with a total of 51 points), which has been validated as a prognostic tool and intermediate judgment criterion for the diffuse cutaneous form (Appendix 1).

- Trophic disorders

- *Digital ulcers*—these can be:

- of ischemic origin, affecting the ends of the fingers (the pulp most often), which can progress to necrosis or gangrene;
- of mechanical or traumatic origin, with an extruding calcinosis or a pressure point against a bony prominence.

- Pulp scarring, which sometimes persists after the healing of an ulcer.

- Calcinoses: they usually occur in soft tissues, most frequently at the fingertips. They should also be looked for on the extensor surfaces of the forearms or on the front side of the knees. They exist in very extensive forms, affecting the periarticular or articular areas, muscles, and tensions, which are difficult to treat. They can progress towards fistulization, extrusion of chalky substance, and formation of sores that are often painful and long lasting.
- Telangiectasias: when present, they most frequently occur on the hands, face, and lips, and in the mouth.
- Pigmentation disorders: areas of hyperpigmentation or depigmentation can be observed. Sometimes, a melanodermal appearance is found.
- Articular and musculotendinous signs

- Articular damage: most frequently this is inflammatory arthralgias and stiffness of the fingers, hands, and wrists. Less frequently, arthritides are observed: approximately 10–20% of patients have clinical synovitis during the course of their illness. Significant joint deformities can occur that, compounded with dermatological involvement, can lead to complex and debilitating deformities.
- Tenosynovial involvement: this leads to tendon friction rub, which is a marker of the severity of the disease. In severe forms, irreducible retraction of the fingers may occur. Tenosynovial involvement can cause carpal tunnel syndrome.
- Muscular damage: this ranges from simple myalgias to a proximal motor deficit with extremely variable prevalence based on the criteria used (clinical, biological, electromyographic, MRI, and/or histological criteria).
- Bone damage: bone resorption of acroosteolysis type must be looked for. It mainly affects the distal phalanges of the hands and feet, but can also involve other sites. There also exists an increased risk of osteopenia and osteoporosis.

## Screening for visceral damage

The physical examination will then look for indications of visceral damage from SSc:

- Pulmonary damage
  Any dyspnea should be looked for:
  - Diffuse infiltrative lung disease (ILD): ILD is most frequently asymptomatic at first. It should be considered in case of persistent dry cough or dyspnea, which should be looked for in any patient with SSc. The presence of dry basal crackles must be systematically looked for in all patients. If ILD is detected, an etiological assessment must be conducted in order not to mistakenly attribute ILD to the SSc and in particular to look for the use of a potentially involved drug (e.g., amiodarone).
  - PAH: this should be systematically considered in presence of asthenia, fatigue on exertion, palpitations, dyspnea on exertion, or signs of right heart decompensation (edema of lower limbs, jugular venous distention, hepatojugular reflux). PAH can also manifest as chest pain and oppression, lipothymias and syncopes (sign of severity), and very rarely by hemoptysis. A systolic murmur of tricuspid regurgitation and/or loud S2 in the pulmonary area should be looked for. Identification of PAH can precede diagnosis of SSc.
  - Other causes of dyspnea are of course possible, particularly left ventricular failure, pulmonary embolism, anemia, respiratory tract infection, neoplasia, thyroid disease, respiratory muscle dysfunction in the context of an associated inflammatory myopathy, and peripheral muscle weakness.
  - It is not uncommon for multiple pathologies to coexist in the same patient and contribute to dyspnea.

- Cardiac damage
  The clinical signs indicating possible cardiac damage are the following: signs of left and/or right heart failure, palpitations, lipothymia, syncope, chest pain, rhythm, and/or conduction disorders on ECG.
- Renal damage
  The clinical signs indicating a possible scleroderma renal crisis are the following: those of HBP (although normotensive forms of scleroderma renal crisis are possible), sometimes a malignant HBP and/or oliguria, proteinuria, signs of kidney failure, and signs of thrombotic microangiopathy. Renal crisis can be an indication of SSc.

The predictive factors of the occurrence of a scleroderma renal crisis are summarized in Table 2.

**Table 2:** Predictive factors of occurrence of scleroderma renal crisis

Diffuse skin damage
Rapid progression of skin damage
Duration of disease progression < 3–5 years
Recent cardiac event
Pericarditis
Left ventricular failure
Anemia of recent onset
Anti-RNA polymerase III antibodies
Prednisone treatment > 15 mg/day within the last 3 months

Adapted according to Steen 2003

• Orofacial involvement

There are numerous orofacial manifestations of SSc that lead to functional and/or tissue damage. Oral iatrogenic manifestations related to treatments used are also reported.

- *Functional damage*: Masticatory difficulties due to a limitation of the buccal opening (less than 40 mm between the dental arches) by cutaneous and labial sclerosis associated with a limitation of the mandible’s excursion movements (opening, protrusion, right and left lateral).
- *Neuropathic damage*: Some patients can have neuropathic pain, especially from serious trigeminal neuralgias, set off by stimulation of a trigger zone, as well as from stomatodynia. Joint and mandibular pain, such as headache and myalgia during chewing, have also been reported in literature, frequently associated with dysgeusia and dysphagia.
- *Bone and joint damage*: In these patients, a protrusion of the anterior part of the lower portion of the face has been noted, along with anomalies in the temporomandibular joints as well as skeletal changes in the face. SSc can also lead to idiopathic mandibular bone loss/resorption. The occurrence of mandibular resorption and temporomandibular joint damage, sometimes by calcinosis, can result in limitations in the opening and closing of the mouth, along with dental occlusion anomalies.
- *Mucosal damage*: There is fibrosis of the mucosae and more particularly of the gums, predisposing to the risk of periodontal disease. The inflammation inherent to scleroderma resulting in periodontal vascularization defects has also been reported. Added to this is hyposialia, due in part to fibrosis of the salivary glands, and sometimes amplified by mouth breathing in connection with lip incompetence. This reduction in saliva flow creates a stagnation of cariogenic bacteria and periodontal pathogens. Thus, the increase in the incidence of periodontal disease is related to different factors compounded by increased difficulties in oral hygiene in SSc patients (limited buccal opening, microstomia, jugal tissue tensions).
- *Dental damage*: The Individual Caries Risk (ICR) is also increased in these patients, linked in part to the hyposialia but also to the frequent presence of GERD, leading to a reduction in salivary pH and resulting in carious lesions and dental erosions.
- *Iatrogenic damage*: Besides pathology, certain SSc treatments can lead to adverse effects in the orofacial area and require special precautions during dental care.

- Long-term immunosuppressants noticeably increase the risk of infection and require doses of antibiotic prophylaxis for invasive orodental procedures. They are also a factor favoring the appearance of oral candidiasis, especially in the context of hyposialia.
- Furthermore, ulcerations of the oral cavity can occur when methotrexate (MTX) treatment is started. Adapted treatment will then be ordered to treat the oral ulcerations, often requiring a reduction of the prescribed dose.
- Prescription of anti-bone resorption agents, such as bisphosphonates or denosumab to prevent steroid-induced osteoporosis, can cause osteochemonecrosis of the jaw. Buccodental assessment before treatment, along with routine checkups, will then be necessary.
- Patients with an associated Raynaud’s phenomenon are treated with calcium channel blockers that are sometimes known to cause gingival hypertrophy. It is therefore advised to propose that patients have regular periodontal checkups.

Digestive damage

- Esophageal damage: This can appear with dysphagia, odynophagia, retrosternal pain (secondary to esophageal motility disorders or esophagitis), gastroesophageal reflux along with possible complications (esophagitis, peptic stricture, Barrett’s esophagus).
- Gastric damage: This can appear with a dyspeptic syndrome, anorexia, early satiety, and even total food intolerance, suggestive of gastroparesis or at most a bezoar fostered by gastric atony. A digestive hemorrhage can result in a “watermelon” stomach (gastric antral vascular ectasia).
- Intestinal damage: The alerting symptoms are nonspecific, e.g., abdominal discomfort, bloating, nausea and/or vomiting, transit problems.
- Malabsorption syndrome with a microbial overgrowth syndrome can be indicated by a change in general condition (weight loss) and diarrhea associated with a clinical and/or biological deficiency syndrome.
- Intestinal pseudo-obstruction disorder can be indicated by crises of diffuse abdominal pain, constipation, and meteorism of variable intensity occurring intermittently, creating a picture of repetitive subocclusive or even occlusive syndrome (chronic intestinal pseudo-obstruction, CIP).
- Colonic damage: The main clinical sign is constipation (≤ 2 spontaneous stools weekly) associated with abdominal meteorism, potentially resulting in an occlusive syndrome related to the formation of fecalomas.
- Anorectal damage: Fecal incontinence, rectal prolapse.
- These digestive disorders, when they occur, can cause malnutrition which requires monitoring for weight loss, calculation of the body mass index (BMI), and albuminemia testing. The diagnosis of malnutrition will be considered in the presence of one or more of the following criteria:
- Weight loss ≥ 5% in 1 month or ≥ 10% in 6 months
- BMI < 21 kg/m^2^
- Albuminemia < 35 g/l
- **Urogenital involvement**

Look for a small scleroderma bladder (suggested by pollakiuria); in men, erectile dysfunction; in women, urinary incontinence, which does not seem rare and is probably underestimated, dyspareunia, vaginal dryness.

- **Neuropsychiatric manifestations**

Involvement of the central nervous system is exceptional, but mood disorders including depression and cognitive disorders are more frequent than in the general population.

- **Screening for another associated disease**
  SSc is associated with another autoimmune disease in a quarter of all cases.
- Thyroid disease: Clinical involvement of the thyroid is rare (less than 5% of cases). Most frequently, it is autoimmune hypothyroidism (Hashimoto’s disease).
- Sjögren’s syndrome: A syndrome of ocular or oral dryness is present in one- to two-thirds of patients. An associated Sjögren’s syndrome meeting American–European criteria is found in about 10% of cases.
- Primary biliary cholangitis: Look for pruritus or jaundice. It occurs in less than 5% of SSc patients. This association is called Reynolds syndrome. The frequency of Sjögren’s syndrome is then even higher. SSc can also be associated with systemic lupus, rheumatoid arthritis, and inflammatory myopathy or appear during the progression of a mixed connective tissue disorder (or Sharp syndrome).
- Cancer: An associated cancer must be looked for in case of significant change to the general condition, in SSc occurring after 60 years of age, especially in cases of diffuse cutaneous SSc with anti-RNA polymerase III antibodies, where cancer is sometimes synchronous.

### Paraclinical examinations

Paraclinical examinations allow:

- Confirmation of the diagnosis of SSc when clinical signs are insufficient
- The search for visceral complications

#### Examinations permitting confirmation of the diagnosis of SSc

Autoantibody testing and periungual capillaroscopy are additional first-line examinations conducted when SSc is suspected.

- Autoantibody testing
- Antinuclear antibodies (ANA) by indirect immunofluorescence on HEp-2 cells: Most patients (> 90%) have ANA at titer > 1/160 or > 1/200. Two main types of fluorescence are observed: anticentromere appearance or nucleolar appearance. However, in 20–30% of cases, no target antigen is identified. For patients without ANA or without an identified specificity, retesting at a later time is useful, because they may be positive secondarily or express a specificity secondarily.
- Main ANAs found with SSc (usually exclusively):
- Anticentromere antibodies defined by the appearance of fluorescence.
- Antitopoisomerase I (anti-Scl70) antibodies. Their identification relies on immunoblot.
- Anti-RNA polymerase III antibodies, associated with an elevated risk of scleroderma renal crisis. Their identification relies on ELISA or immunoblot, depending on the laboratory.
- Anti-U1RNP antibodies, which can be found in SSc. These antibodies are not specific to SSc, being also present in Sharp syndrome (mixed connective tissue disease) and other forms of overlap.
- Antibodies of scleroderma–myositis overlap syndromes: Anti PM-Scl antibodies, anti-Ku antibodies. Their identification relies on immunoblot.
- Scleroderma-Dot can detect the presence of other, usually exclusive, specific antibodies such as anti-fibrillarin and anti-Th/To antibodies.
  Identification of the specific antibody allows identification of the SSc forms more at risk for certain visceral complications. However, these associations are neither automatic nor exclusive. For example, one patient with anti-Scl70 may have no visceral, especially pulmonary, involvement, while another patient with anticentromere could have severe ILD (Table 3).

**Table 3:** Antinuclear antibodies associated with systemic sclerosis (SSc)

Antinuclear antibody specificities	Appearance of fluorescence	% of patients	Form of the disease	Clinical phenotype
Anticentromere antibodies	Centromeric	20–40%	Limited cutaneous SSc	PAH
				Severe peripheral vascular disease
Anti-Scl-70 or anti-topoisomerase antibodies	Homogeneous and nucleolar	20–30%	Diffuse cutaneous SSc	Severe ILD
				Severe peripheral vascular disease
Anti-RNA polymerase III	Speckled (±nucleolar)	20–25% in Caucasians of North America and the UK and about 5% in France, Germany, Italy, and Japan	Diffuse cutaneous SSc	Rapidly progressive skin damage
				Scleroderma renal crisis
				Cancer
Anti-U3-RNP (antifibrillarin)	Nucleolar	In 4–10%	Diffuse cutaneous SSc > Limited cutaneous SSc	ILD
				PAH
				Renal crisis
				Digestive damage of small intestine
Anti-Th/To	Nucleolar	2–5%	Limited cutaneous SSc	ILD
				PAH
Anti-Pm-Scl	Speckled and nucleolar	2%	Limited cutaneous SSc (overlap with myositis)	Myositis
Anti-U1 RNP	Speckled		Limited cutaneous SSc (overlap with MCTD)	Arthritis
				Myositis
				PAH
Anti-U11/U12 RNP	Speckled	1–3%	Diffuse cutaneous SSc/limited cutaneous SSc	Severe ILD
Anti-Ku	Speckled		Limited cutaneous SSc (overlap syndrome with myositis)	Myositis
Anti-RuvBL 1/2	Speckled	1–3%	Diffuse cutaneous SSc (overlap myositis)	Myositis

*Ab* antibody, *PAH* pulmonary arterial hypertension, *ILD* interstitial lung disease, *RNA* ribonucleic acid, *RNP* ribonucleoprotein, *MCTD* mixed connective tissue disease
- Periungual capillaroscopy
  This examination allows looking for organic microangiopathy. Only the presence of megacapillaries and capillary rarefaction are characteristic (although not completely specific) elements of SSc. The sclerodermal landscape in the capillaroscopy is defined by the existence of megacapillaries, rarefaction, and disorganization of the loops. It is only observed in systemic sclerosis and certain connective tissue diseases (dermatomyositis and in mixed connective tissue disease). This organic microangiopathy can take on three main appearances as described by Cutolo, without being exclusive because different “stages” can appear in the same patient:
- Early stage: presence of megacapillaries
- Active stage: reduction in the number of capillaries, presence of megacapillaries with hemorrhage
- Late stage: reduction in the number of capillaries with barren zones, neovascularization
  However, these different stages do not constitute stages of severity or progression of scleroderma.
- Skin biopsy is not recommended for SSc diagnosis.

#### Other biological examinations necessary during the initial assessment

- CBC-platelets
- Reticulocytes, schizocytes, haptoglobin, LDH in case of suspected scleroderma renal crisis
- Blood electrolytes, creatinine, uric acid, CRP
- Fasting blood glucose, calcium, phosphorus
- Albumin, hepatic assessment (AST, ALT, γGT, total bilirubin, and alkaline phosphatases)
- CPK
- Urine strip (possibly urine culture and protein-to-creatinine ratio on a sample)
- NT-proBNP (or BNP)
- TSH
- Ferritin level

#### Paraclinical examinations for detecting visceral complication

Overlap myopathy associated with systemic sclerosis

- CPK and possibly aldolase tests.
- Based on the clinical symptomatology, electromyogram, muscle MRI
- Muscle biopsy may be necessary in case of myolysis with muscle deficit. Patients with overlap myopathy associated with scleroderma have anti-PM/Scl antibodies in 50% of cases.

Osteoarticular damage

- In destructive polyarthritides, the association with rheumatoid arthritis should be sought with tests for anticitrulline cyclic peptide (CCP) antibody and rheumatoid factor.
- X-rays of the hands looking for acroosteolysis, articular erosions or impingements, and/or calcinotic lesions.
- Other x-rays may be proposed based on the symptomatology.
- In some cases, a lesion assessment of arthritis and synovitis can be done by ultrasound to analyze possible inflammatory components with Doppler or even articular MRI, or sometimes a CT scan, especially if a surgical procedure is planned.
- Bone densitometry can aid in screening for possible osteoporosis.

Interstitial lung disease

- High-resolution CT scan
- Pulmonary function tests (PFT) with measurement of lung volumes, in particular FVC (spirometry) and total lung capacity (TLC) (spirometry or plethysmography), measurement of the FEV1/VC ratio, and measurement of cDLCO.
- High-resolution chest CT scan with thin slices, systematically in all patients regardless of form of SSc and serological status.
- In the presence of ILD, a 6-min walk test with Borg Dyspnea Scale and saturation test is recommended by the working group. Although not validated for SSc, it constitutes a good evaluation with moderate effort. Bronchoalveolar lavage (BAL) has no diagnostic benefit but can still be proposed in certain situations (screening for complications, particularly infections, alveolar hemorrhage).
- Lung biopsy: There is no indication for performing lung biopsy in patients with ILD from SSc apart from very rare, particular cases.

Pulmonary hypertension (PH)

- Annual screening for PH is recommended by echocardiogram and PFT with DLCO, with special attention to patients with DLCO < 60% without or with limited ILD.

Biological tests

- BNP or NT-proBNP tests
- Arterial blood gas test after an Allen test to eliminate cubital vein thrombosis
- Blood uric acid (hyperuricemia being a predictive marker for PAH in the DETECT score)

Technical procedures

- ECG
- PFT with measurement of cDLCO
- Doppler echocardiography.
- In case of suspected PH: right heart catherization
- High-resolution chest CT scan
- Lung scintigraphy of ventilation and perfusion, or even pulmonary angiogram

**Fig. 3:**
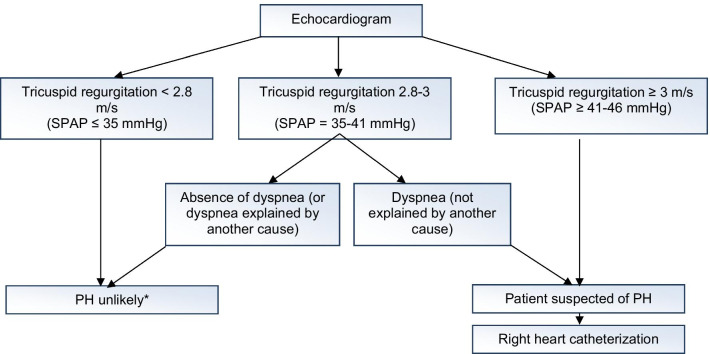
Algorithm for PAH screening in SSc. *SPAP* systolic pulmonary artery pressure estimated according to Bernoulli’s formula: V_IT_^2^ + P_OD_ estimated, *PH* pulmonary hypertension, *SPAP* systolic pulmonary artery pressure, *PH* pulmonary hypertension, *VIT* tricuspid regurgitation velocity, *RAP* right atrial pressure. *In absence of indirect signs of PH, no dilation of the right cavities, no shortening of the pulmonary acceleration time. Special attention must be given to patients with a cDLCO < 60%, especially for patients with a disease progression of more than 3 years. The DETECT algorithm can then be useful to identify patients at risk for PAH (http://detect-pah.com). The decision to perform a right heart catheterization requires multidisciplinary coordination with cardiologists and pneumologists.

In practice, measurement of tricuspid regurgitation permits assessment of SPAP, and according to the algorithm below, it is possible to discuss a potential right heart catheterization (Fig. 3).

Cardiac damage

Biological tests

- Natriuretic peptides (BNP or NT-proBNP) in case of confirmed or suspected left or right ventricular dysfunction, in cases of PAH.
- Troponin in case of suspected coronary ischemia or myocarditis

Morphological examinations and technical procedures

- ECG
- 24-h Holter ECG in case of palpitations, lipothymia, or syncope
- Doppler echocardiography
- Heart MRI can be proposed in case of clinical or paraclinical symptoms to especially differentiate inflammatory damage (myocarditis) from fibrosing damage (primary heart damage), but it should not be systematic (frequent subclinical lesions of uncertain prognosis).

Scleroderma renal crisis

Biological tests

- Creatinine, plasma electrolytes, urea
- Blood smear with search for schizocytes and tests for reticulocytes, LDH, free bilirubin, haptoglobin
- 24-h proteinuria or proteinuria/creatininuria on a sample
- Urine culture (seeking leukocyturia, hematuria) (note that hematuria with a strip can correspond to hemoglobinuria in a hemolytic situation and not to hematuria)
- In the presence of microscopic hematuria and in the absence of HBP, an ANCA test will be performed (rare associations of SSc and MPO-ANCA-associated vasculitis).
- In certain cases of scleroderma renal crisis with a picture of thrombotic microangiopathy, a blood test for the ADAMTS13 protein can be done to eliminate an exceptional associated PTT (in scleroderma renal crisis, no ADAMST13 deficiency, no anti-ADAMTS13 antibodies).

Morphological examinations and technical procedures

- Renal ultrasound and, based on context, Doppler ultrasound of the renal arteries (to eliminate associated renal artery stenosis). Renal biopsy: Renal biopsy is not needed to diagnose a scleroderma renal crisis and in all cases will only be done after normalization of the arterial pressure. On the other hand, it should be done for atypical forms.

The diagnosis of scleroderma renal crisis can be established using the criteria proposed in Table 4 (international criteria are in the process of being defined).

**Table 4:** Classification criteria for scleroderma renal crisis

(a) Hypertensive forms	
a. HBP ≥ 140/90 mmHg (or increase of systolic BP ≥ 30 mmHg or diastolic BP ≥ 20 mmHg) obtained on two different measurements with a minimum separation of 5 min, with no explanation other than SSc	
b. Acute renal injury, with no explanation other than SSc AKI according to the KDIGO classification: more than 50% increase of serum creatinine from the reference value within the preceding 7 days or an absolute increase of 26.5 µmol/l (≥ 0.3 mg/dl) in 48 h	
c. Thrombotic microangiopathy	
New or aggravated anemia without other explanation	
Schizocytes	
Thrombopenia ≤ 100,000/mm^3^ confirmed on a smear	
Signs of hemolysis: elevated LDH, low haptoglobin, increased reticulocytes	
Negative antiglobulin test	
d. Target organ dysfunction	
Hypertensive retinopathy	
Hypertensive encephalopathy	
Pulmonary edema	
Acute pericarditis	
e. Anomalies suggestive of renal biopsy (fibrinous thrombi, fibrinoid necrosis, glomerular collapse, onion bulb proliferation in pre-glomerular arterioles and arch arteries)	

(b) Normotensive forms	
1. Increase of creatinine level > 50% of baseline value OR creatinine level ≥ 120% of upper normal laboratory reference value AND 2. At least one of the following five criteria: a. Proteinuria ≥ 2+ by strip b. Hematuria ≥ 2+ by strip or ≥ 10 RBC per field c. Thrombopenia < 100,000/mm^3^ d. Hemolysis defined by anemia not linked to another cause with: (1) Schizocytes or other RBC fragments found on blood smear (2) Increase in reticulocyte level e. Renal biopsy showing a typical appearance of scleroderma renal crisis (fibrinous thrombi, fibrinoid necrosis, glomerular collapse, onion bulb proliferation in pre-glomerular arterioles and arch arteries)	

*HBP* high blood pressure, *BP* blood pressure, *SSc* systemic sclerosis, *AKI* acute kidney injury, *LDH* lactate dehydrogenase

Digestive damage

Biological tests

- Hemoglobin test
- Ferritin level
- Vitamin B9 and B12 tests: in case of macrocytic non-regenerative anemia
- Tests for fat-soluble vitamins A, D, E, K, and B1 (thiamin) in case of small intestine damage with microbial overgrowth
- Electrophoresis of serum proteins
- Prealbumin in case of suspected malnutrition

Morphological examinations and technical procedures

- Esophageal manometry: Although its benefit has not been demonstrated, it is performed by some teams during the initial assessment, especially if the SSc diagnosis is doubtful. Esophageal manometry is also used to screen for esophageal motility disorders and assess their severity, which can facilitate explaining certain symptoms such as dysphagia or reflux.
- Esophagogastroduodenoscopy
- This is performed for diagnostic purposes in case of persistent upper digestive symptoms, to screen for Barrett’s esophagus, to look for peptic esophagitis, gastroduodenal telangiectasias, or even an authentic watermelon stomach (gastric antral vascular ectasia).
- It will also be done in patients who have digestive symptoms or progressing iron-deficiency anemia.
- Video capsule (on specialist’s recommendation) if iron deficiency anemia is unexplained (upper and lower digestive fibroscopy normal and without gynecological cause) to look for bleeding from intestinal telangiectasias.
- Measurements of pH and impedance (on specialist’s recommendation)

Malabsorption syndrome and chronic intestinal pseudo-obstruction (CIP)

1. Glucose breath test: used to screen for microbial overgrowth
2. Small bowel transit or CT enterography or MRI enterography on specialist’s recommendation
3. Small bowel manometry can be performed on a specialist’s recommendation but is only available at some specialized centers.

Colonic motility disorders or colonic mucosal anomalies

- An abdominal CT scan in case of occlusive syndrome or suspected cystic pneumatosis
- Colonoscopy should be performed in case of suspected lower digestive bleeding

Anorectal involvement

Patients with fecal incontinence will have:

- A proctological examination
- Anorectal manometry and endorectal ultrasound in case of fecal incontinence without rectal prolapse, to be able to propose anorectal biofeedback training

#### Examinations for detecting an associated disease

These examinations will be performed based on clinical and/or biological context.

Hypothyroidism from Hashimoto’s disease

- TSH and FT4 test
- In case of hypothyroidism: anti-thyroid peroxidase antibodies
- Possibly supplemented by thyroid ultrasound

Sjögren’s syndrome

- Anti-Ro/SSA 52 KDa and 60 KDa antibodies, rheumatoid factors
- Nonstimulated salivary deficiency
- Minor salivary gland biopsy
- Parotid gland ultrasound in case of parotid enlargement
- Ophthalmological examination with Schirmer’s test
- Gynecological examination

Primary biliary cholangitis

- Liver function test
- Anti-mitochondrial M2 antibodies, anti-gp210 antibodies
- Hepatobiliary ultrasound
- Bili-MRI
- Second-line hepatic needle biopsy

**Other systemic diseases** can be associated much more rarely, such as systemic lupus (anti-native DNA Ab, anti-Sm Ab, complement), antiphospholipid syndrome (anticardiolipin Ab, circulating anticoagulant), rheumatoid arthritis (anti-CCP Ab), or autoimmune myositis (anti-JO1 or other anti-synthetase Ab in particular).

### SSc impact assessment

At the time of SSc diagnosis, it is indispensable to assess the functional and psychological impact of the disease. Everyday functional discomfort, especially in the fingers and hands, must be assessed. It is also necessary to assess the professional (or educational, in children), social, and family impact of SSc, and listen to patients and their family and friends when giving the diagnosis. Assessment scales are useful, especially in clinical research, such as the SHAQ for scleroderma or the SF-36 scales, the Cochin Hand Functional Scale (Appendix 2). In children, stature and pubertal growth must be monitored.

## Therapeutic management

### Objectives

The absence of global treatment acting simultaneously on each of the different pathogenic mechanisms of SSc makes therapeutic management particularly difficult.

Treatment of visceral damage is the essential objective. It is based on the type and severity of visceral damage (Table 5).

**Table 5:** Systemic sclerosis treatments based on affected organ

Manifestations	Treatment
Peripheral vascular damage	Calcium channel blockers Prostacyclin analogs Endothelin A and B receptor antagonists: bosentan (prevention of occurrence of new digital ulcers) Phosphodiesterase type 5 inhibitors: sildenafil (healing of digital ulcers) Platelet aggregation inhibitors in case of macroangiopathy
Skin damage	Methotrexate MMF Cyclophosphamide Therapeutic intensification and autologous hematopoietic stem cell graft in case of severe and progressive diffuse form after validation of indication in MCM (see Appendix 4)
Articular damage	Nonsteroidal antiinflammatory drugs if no upper digestive damage Low-dose corticosteroids (≤ 10 mg/day) Methotrexate Leflunomide Targeted biologic treatments only in case of refractory arthritis: abatacept, rituximab, tocilizumab
Inflammatory myopathy	Oral corticosteroid therapy Methotrexate Intravenous immunoglobulins
Diffuse infiltrative lung disease	MMF Intravenous cyclophosphamide followed by azathioprine or MMF Low-dose corticosteroid therapy (10–15 mg/day) (discussed) Rituximab Nintedanib Oxygen therapy Lung transplant
Pulmonary arterial hypertension	Oxygen therapy Diuretics Endothelin receptor antagonists: bosentan, ambrisentan Phosphodiesterase 5 inhibitors: sildenafil, tadalafil Prostacyclin receptor agonists: selexipag Prostacyclin analogs: epoprostenol, treprostinil Atrial septostomy Lung or heart–lung transplant
Heart	Calcium channel blockers Angiotensin II converting enzyme inhibitors or angiotensin II receptor blockers or neprilysin inhibitors and angiotensin II receptor blockers, Beta-blockers Diuretics–mineralocorticoid receptor antagonists Antiarrhythmia drugs Implantable defibrillator/stimulator Sometimes immunosuppressants if myocarditis Heart transplant
Renal Crisis	Angiotensin-converting enzyme inhibitors Intravenous calcium channel blockers Dialysis Kidney transplant
Digestive damage	Esophagus: proton pump inhibitors, prokinetics (metoclopramide, domperidone) Stomach: proton pump inhibitors, erythromycin (125–250 mg × 2/day), clavulanic acid, prokinetic (metoclopramide, metopimazine) Small bowel: in case of motility disorders and/or intestinal pseudo-obstruction, octreotide (50–100 µg/day) Colon: in case of constipation, balanced diet with fibers and mucilages, adequate hydration, regular physical activities, laxatives and cleansing enemas, prokinetics for a limited period (metoclopramide, domperidone) Enteral and parenteral feeding: in case of severe small bowel damage or deglutition disorders Small intestinal bacterial overgrowth: sequential antibiotic therapy (three antibiotics among amoxicillin, metronidazole, fluoroquinolones, gentamycin, etc.)

*MCM* multidisciplinary coordination meeting, *MMF* mycophenolate mofetil

The treatment objectives are:

- To limit or stop the disease’s progression
- To reduce sequelae
- To improve the patient’s quality of life by managing the disability and loss of function

### Professionals involved

Therapeutic treatment is multidisciplinary and coordinated by the general physician in connection with specialists and hospital physicians from a reference and/or competence center.

Specialists most frequently involved:

- Internists, rheumatologists, dermatologists, pediatricians, cardiologists, pulmonologists, gastroenterologists, vascular specialists, nephrologists, functional rehabilitation specialists, stomatologists, occupational therapists, nutritionists, or dietitians
- Psychologist, psychiatrist
- Any other specialist whose opinion is necessary based on the clinical profile

### Therapeutic education and lifestyle change

Therapeutic patient education (TPE) is an important part of the management of a chronic disease and a key element in the overall management of the patient. This approach, which must be multidisciplinary, has been defined by the WHO as follows:

“TPE enables patients to acquire and maintain the skills they need to better manage their lives with a chronic illness.

It is an integral and permanent part of patient care; it includes organized activities, including psychosocial support, designed to make patients aware and informed of their disease, care, hospital organization and procedures, and health- and disease-related behaviors. This is to help them (and their families) understand their disease and their treatment, work together and assume their responsibilities in their own care, in order to help them maintain and improve the quality of their lives.

Oral or written information and prevention advice can be provided by a health professional on various occasions, but this is not the same as therapeutic education of the patient.”

“The educational approach requires active participation and is centered on the person and not on the simple transmission of knowledge and skills.”

"It is a partnership between the patient, their family and friends and the healthcare team, with the goal of helping the sick person take care of him or herself.”

• TPE goals

TPE contributes to improving the patient’s health and quality of life, along with those of their circle of family and friends.

The specific goals of TPE are:

- Acquisition and maintenance of self-care skills by the patient
- To relieve symptoms
- To take into account the results of self-monitoring and self-measurement, adapting doses of medication
- To perform technical and care procedures
- To implement lifestyle changes (balanced diet, physical activity, etc.)
- To prevent avoidable complications
- To deal with problems caused by the disease
- To involve his/her social circle in managing the disease, treatments, and their repercussions
- Implementation or acquisition of adaptation skills
- To know oneself, and have confidence in oneself
- To know how to control one’s emotions and master stress
- To develop creative reasoning and critical reflection
- To develop communication and interpersonal skills
- To make decisions and resolve problems
- To set goals and make choices
- To observe, evaluate, and strengthen oneself

• Information and therapeutic education do not share the same goals.

The first consists of delivering information to a “passive” patient. It is part of the duties of all physicians and is a legal right of the patient (Law of March 4, 2002).

The “educational” dimension goes farther, because benefiting from information on the disease does not mean learning to live with it. TPE is based on an “active” attitude of a patient who questions, reacts, expresses him/herself, and dialogues with healthcare professionals and/or with peers. Every person is different, and every situation unique. This personalized and caring “accompaniment” helps the patient to make decisions, sometimes difficult and complicated, about care, so as to improve his/her quality of life and therefore that of his/her family and friends. It also helps the patient with choices concerning his/her life plan, orientation, administrative matters, etc.

• TPE includes four steps:

The HAS (Haute Autorité de Santé, or High Health Authority) has issued recommendation guidelines to assist in the implementation of educational programs or steps.

1. Drafting of an individualized educational diagnosis (shared educational interview) with the patient that allows him/her to define his/her needs, expectations, fears, beliefs, plans, etc.
2. Definition of a personal TPE program that defines the “skills” (self-care and adaptation knowhow) that the patient can acquire and/or practice
3. Planning and implementation of TPE sessions that require highly codified content and learning methods
4. Evaluation of the learning at the end of the educational program (individual evaluation of the “skills”)

• TPE in practice: three operational modalities

These are essentially:

1. TPE programs whose approach is medical, High Health Authority (HAS) approved, and conform to a national specification whose modalities of implementation and content are defined by order of the Minister of Health. These programs are implemented locally following the authorization of the regional health agencies (RHA). They include the patient’s daily life and social, psychological, and environmental factors. They are based on scientific information (professional recommendations, relevant scientific literature, professional consensus) and are enriched by feedback from patients, their circle of family and friends, and patient associations, in terms of content and educational resources. They are organized by a multidisciplinary healthcare team trained in TPE and peers (involved patients, TPE experts, and members of patient associations);
2. Learning programs, aimed at patients’ acquisition of technical procedures allowing the use of a medication
3. Care actions aimed at providing assistance and support to patients, or their circle, in the management of a chronic disease

• TPE for scleroderma patients

**Table:** 

Things to consider	Pedagogical objectives in terms of skills for the patient to acquire (list not exhaustive)
What is scleroderma?	Be able to explain yourself and in your own words to those around you the mechanism of the disease (chronic nature, autoimmune mechanisms, daily manifestations, etc.) Give meaning to observing medical follow-up. The patient must have available information on routine disease monitoring exams, in order to understand their need and importance, and to adhere to plans when applicable and/or to anticipate and prepare for future appointments and consultations Develop and/or maintain appropriate actions when signs suggestive of an aggravation of his/her state of health appear
Prevention of Raynaud’s syndrome, digital ulcers	Determine and organize the patient’s active role in the management of Raynaud’s phenomenon prevention: Identify and anticipate at-risk situations and establish preventive measures (protected exposure to cold, humidity, wind, quitting smoking, protection from trauma, etc.); Develop appropriate actions to handle the appearance of digital ulcers; Discover and learn material and technical aids in terms of prevention and assistance in daily life
Living with	Help the patient to: Express his/her experience of life with the disease, his/her own representations, his/her own sufferings Clarify his/her own feelings; seek special treatment when necessary; psychologist, psychiatrist Identify personal and external resources to use when confronting difficulties Discover new helpful resources; plan personal strategies Let oneself plan projects to live better with the disease Manage fatigue better Adopt self-care measures centered on his/her wellbeing and comfort in order to reinforce/improve his/her self-esteem Meet with a socio-esthetician to talk about beauty care and camouflage (telangiectasias) Talk about the disease with loved ones (family caregivers) Recognize the difficulties of loved ones (family caregivers) facing scleroderma (positive or negative impact on loved ones) Take care of one’s loved ones (family caregivers): understand chronic stress, fatigue, avoid exhaustion, etc.
Treatments	Help the patient to optimize the management of his/her treatment over the long term: know how it works, its possible adverse effects, the risks of stopping it too soon, rules for monitoring and adaptation Managing one’s treatment in certain situations of daily life (work, going out, vacations, etc.) Importance of hygiene rules in case of immunosuppressant treatment (awareness of vaccination schedule, avoiding potential infectious contacts, etc.)
Digestive damage Hygieno-dietary measures	Allow the patient to clarify his/her own knowledge of the digestive damage of scleroderma Speak to the patient of anorectal digestive disorders in order to be able to express his/her difficulties rarely discussed spontaneously in consultation Implement lifestyle changes in terms of → hygieno-dietary measures: Adapt food, meals; Adopt a healthy and balanced diet; Manage the most frequent digestive disorders; Prevent and treat constipation and lower gastrointestinal dysmotility → prevention of gastroesophageal reflux disease
Pulmonary damage	Allow the patient to understand and explain the pulmonary damage of scleroderma Identify the signs or symptoms suggestive of pulmonary damage; Give meaning to medical monitoring by identifying the specific examinations for diagnosis and monitoring of pulmonary damage; Identify treatment strategies implemented when pulmonary damage occurs

Patients who have already completed one or more TPE days can also participate in personalized or group workshops by appointment (sophrology, psychomotricity, art therapy, etc.) to take a step back from the disease.

### TPE for loved ones, “family or friend caregivers” who support scleroderma patients

The role of caregivers is essential for supporting patients with SSc. For the loved one (family caregiver), seeing the other who is not in good health reflects back on their own fragility and vulnerability. And the caregiver’s own life can be disturbed (personal life, relationships, professional life, etc.) by the pressure of helping (subjective and/or objective “burden”).

It is necessary to prevent, identify, guide, and take care of the needs and difficulties associated with this support. Recognition and specific hospital support for family caregivers should improve the quality of the help provided by family caregivers to patients with SSc. It is necessary to follow fragile–vulnerable caregivers, or those with difficulties, and guide them. It is also necessary to offer help and measures adapted to family caregivers who need it: practical advice, specific medical consultations, psychological support, dietary advice, therapeutic hobbies, TPE sessions dedicated to the caregiver, etc.

**Table:** 

^1^ WHO definition 1998: www.euro.who.int/__data/assets/pdf_file/0009/145296/E93849.pdf Report of WHO-Europe, published in 1996, Therapeutic Patient Education – Continuing Education Programmes for Health Care Providers in the Field of Chronic Disease, translated into French in 1998 ^2^ HPST Act: www.has-sante.fr/portail/upload/docs/application/pdf/etp_-_definition_finalites_-_recommandations_juin_2007.pdf ^3^ Alliance Maladies Rares—TPE Methods 2015: www.alliance-maladies-rares.org/sortie-du-1er-guide-complet-de-letp-par-lalliance/ Pages 1–3: understanding TPE and its regulatory framework Pages 4–6: TPE, an opportunity for rare disease associations Pages 7–10: designing a TPE program Pages 11–12: developing support activities Pages 13–14: becoming an “expert” patient/family caregiver Pages 15–16: acquiring skills and training oneself in TPE ^4^Therapeutic patient education guide of the High Health Authority (January 2016): www.has-sante.fr/portail/jcms/c_1241714/fr/education-therapeutique-du-patient-etp ^5^ TPE tools Information on the disease and treatments (scleroderma in 100 questions) www.rhumatismes.net/index.php?p=8&id_bro=14&rub=les100qs Mallette FSMR FAI^2^R—www.fai2r.org	

### Patient associations

All health professions and patients should be informed of the existence of patient associations.

These associations contribute to better overall management of the disease by promoting cooperation between patients, patient associations, and caregivers.

The *Association des Sclérodermiques de France* (ASF, Scleroderma Patient Association of France): http://www.association-sclerodermie.fr), created in 1989, is an association (Law 1901) that was government-approved in 2004. It is made up of scleroderma patients, their spouses, families, friends, and sympathizers, all of whom are concerned and have joined forces to try to conquer scleroderma.

The association was created:

- To help scleroderma patients live better
- To meet and bring together those who are affected by scleroderma
- To share experiences and information about the disease
- To promote medical research

Patient associations and many websites can provide useful information.

### Treatment of specific afflictions and organ damage

For reasons of simplicity, the physicians’ guides cite therapeutic classes in general without listing all of the medications indicated for the pathology concerned.

However, each medication is included only within the precise framework of its marketing authorization (MA). If for explicit reasons this is not the case, and more generally for any prescription of a product outside of marketing authorization (i.e., off-label use), which is done under the sole responsibility of the prescriber, that person must inform the patient and specify that, in this case, he/she may not be reimbursed for the costs incurred for the purchase of the medicine(s) so prescribed. However, most of the drugs used off-label are still covered, on the recommendation of experts or advice of the MCM, as is the case for many rare diseases.

#### Treatment for Raynaud’s phenomenon

Introduction

The severity of Raynaud’s phenomenon in SSc justifies its treatment. No treatment can make it disappear. Treatment is intended to reduce the number of crises, improve quality of life, and prevent digital trophic disorders. Certain drugs are contraindicated or not recommended in case of Raynaud’s phenomenon (Table 6).

**Table 6:** Vasoconstrictor drugs contraindicated or used with precautions for Raynaud’s phenomenon (according to Frances et al. 2008)

Nasal decongestants by local or general administration
Pseudoephedrine
Phenylephrine
Phenylpropanolamine
**Migraine drugs derived from ergot**
Dihydroergotamine
Ergotamine
**Beta-blockers**
**Anti-glaucoma beta-blocker eye drops**
**Hyperprolactinemia treatments**
Bromocriptine
Cabergoline
Lisuride
**Antiparkinsonians**
Pergolide

Two types of medical treatment can be proposed: nonpharmacological and pharmacological.

Nonpharmacological measures

Recommended for all patients:

- Protection from the cold: avoid when possible, wear gloves, use “thermal” clothes which are put on over other clothes to create air pockets and limit the cold, use heaters, heated gloves
- Protection against microtrauma with sometimes the need to adapt workstations
- Quitting smoking, which multiplies the risk of digital trophic disorders by three
- Avoiding vasoconstrictor drugs (Table 6).
  “Alternative” therapies such as acupuncture and biofeedback techniques have not been studied rigorously.

Pharmacological treatments

- **Calcium channel blockers**
  They constitute first-line treatment (nifedipine, diltiazem, nicardipine, nimodipine, amlodipine, felodipine). They significantly reduce the number and severity of bouts of Raynaud’s phenomenon.
  **The only one to have obtained marketing authorization is nifedipine,** which for SSc, at a dosage of 30 mg/day, allows a 30% reduction in the number of crises.
- **Prostacyclin analog**
  Iloprost administered by IV at a dosage of one vial (0.05 mg) per day for 5 days has marketing authorization for “severe Raynaud phenomena with progressive trophic disorders.”
- **Phosphodiesterase 5 inhibitors: sildenafil, tadalafil, vardenafil**
  Several trials have been published which show a modest improvement of Raynaud’s phenomenon in a patient population including scleroderma cases. They are an alternative for severe Raynaud’s phenomenon in case of resistance or intolerance to calcium channel blockers (**off-label use**).
- **Other vasodilators**
  Other than nifedipine, two other products have **marketing authorization** for Raynaud’s phenomenon: moxisylyte hydrochloride and prazosin. Their benefit appears to be modest.
- **Angiotensin receptor blockers**
  Losartan at a dosage of 50 mg/day can be proposed in case of intolerance to calcium channel blockers (**off-label use**).
- **Angiotensin-converting enzyme inhibitors**
  Captopril and enalapril (20 mg/day) can be proposed in case of intolerance to calcium channel blockers or in case of associated PAH (**off-label use**).
- **Serotonin receptor antagonist**

Fluoxetine at 20 mg/day (**off-label use**) is cited in the EULAR 2017 recommendations as an alternative in case of failure or intolerances to vasodilators on the basis of a small randomized trial showing superiority compared with nifedipine.

Surgical treatments

There is no indication for subadventitial thoracic and digital sympathectomy for noncomplicated Raynaud’s phenomenon.

#### Treatment of digital ulcers

Local treatment

The goal of local treatment is to obtain healing and treat secondary infections. It concerns both traumatic and ischemic ulcers.

The principles of treatment are as follows:

- Mechanical debridement of a hyperkeratosis covering an ulcer, of a necrotic or fibrinous base after local anesthesia (5% Emla anesthetic cream (**use within MA**) or 2% Xylocaine gel or 5% nebulizer (**off-label use**)). This debridement must be prudent in the presence of severe ischemia (to be documented by digital pressure measurement). If local anesthesia is insufficient, nitrous oxide can be used (Kalinox or Meopa) (**use within MA**).
- **Cleaning the wound** with physiological serum or soap and water.
- **Occlusive dressings:** hydrogels in case of a dry, fibrinous, or necrotic wound to promote debridement, hydrocolloid, hydrocellular, hydrofiber dressings, or with hyaluronic acid, neutral tulle with Vaseline or paraffin, in granulation phase (Appendix 3).
- **For persistent digital ulcers, surgical debridement and split-thickness skin grafts or full-thickness skin graft can be discussed with competent surgeons** (plastic surgeons, hand surgeons, etc.). Looking for and treating secondary infections are routine.
- **Symptomatic treatment is recommended: analgesics, antibiotics sometimes taken orally, appropriate for bacteriological samples in case of wounds with secondary infections**
  Common wound healing is in general not impaired.

Pharmaceutical treatment of active digital ulcers of ischemic origin

- Calcium channel blockers

Modest efficacy shown in the treatment of Raynaud’s phenomenon in SSc, but there are no data in literature permitting judgment of their possible efficacy in the treatment of digital ulcers. They are, as a rule, frequently already prescribed to patients and will be progressively titrated and maintained at the maximum dose tolerated.

- Prostacyclin analogs

In the majority of cases, pharmaceutical treatment is based on intravenous prostacyclin analogs (Iloprost), although the level of scientific evidence of the drug’s curing efficacy is weak. However, the drug is widely used in the absence of a therapeutic alternative and due to the favorable consensus on its efficacy in severe Raynaud’s phenomenon which is directly responsible for ulcers (**use within MA**).

The therapeutic scheme most often proposed is: Iloprost in syringe pump at dosage of 0.5 to 2 ng/kg/min, adapted to maximum tolerated flow, over 6–8 hours for 5 days. This 5-day cure can later be repeated based on clinical or prolonged response in case of real but incomplete effect.

- Endothelin receptor antagonist: bosentan

This compound has shown a benefit in prevention of digital ulcer relapse in those patients most at risk of multiple digital ulcers (**use within MA**), but it has not shown an effect on the speed of healing ischemic wounds. There is no indication for prescribing bosentan as a curative treatment of digital ulcers.

- Phosphodiesterase 5 inhibitors: sildenafil

Of modest efficacy on Raynaud’s phenomenon, a trial showed a favorable tendency for sildenafil (20 mg, 3 times a day for 3 months) to reduce the healing time for digital ulcers, but it did not achieve statistical significance. On the other hand, the rate of healed ulcers after 3 months was significantly less in the sildenafil group. Sildenafil could be tried on occasion as a second-line treatment in case of insufficient efficacy of or intolerance to iloprost (**off-label use**).

In the absence of favorable progress, an associated cause must be sought (large vessel atheroma, hypothenar hammer syndrome, coagulation disorders, etc.) before any surgical procedure.

**Surgical treatment**

- Amputation

It is necessary to avoid the use of amputation surgery as much as possible. The area of necrosis should be allowed to evolve spontaneously until self-elimination. A limited amputation is sometimes essential in case of wet gangrene or osteitis resistant to medical treatment, to preserve the maximum of healthy tissue, or in case of vast losses of matter without hope of re-epithelialization, or of irreducible distal pain, resistant to analgesics and symptomatic pain treatments.

- Sympathectomy

Periarterial sympathectomy of the digital, intermetacarpal, radial, and ulnar arteries, possibly coupled with microsurgical arterial reconstructions, has no demonstrated efficacy, can result in severe complications in cases of extensive ischemia, and is accompanied by a high rate of recurrence. It is only exceptionally considered as a rescue intervention after failure of medical treatment, in cases of intractable and widespread ischemia.

- Surgical reduction of the volume of calcification

This intervention can be proposed in the event of persistent and painful ulcers with regard to a large calcinosis.

- Total skin debridement–graft

This can be proposed in case of persistent digital ulcers not responding to appropriate local preservative treatments.

**Other therapeutic alternatives**

- The use of cellular therapy techniques by digital injection of stromal vascular fraction or mesenchymal stem cells is still under investigation. These techniques are therefore not recommended currently by the working group.
- Local injections of botulinum toxin do not seem to bring any particular benefit for Raynaud's phenomenon and do not bring any benefit either to healing or to the prevention of recurrence of digital ulcers. They are not recommended by the working group.

**Preventive treatment of ulcer recurrence**

- **Calcium channel blockers**: although their efficacy in the prevention of relapses has not been demonstrated, they are maintained as a treatment for Raynaud’s phenomenon (**use within MA for Raynaud’s phenomenon)**.
- **Prostacyclin analogs**
  Iloprost has been tested in sequential cures in several trials. Currently there is not sufficient evidence in the literature to recommend the use of iloprost in sequential treatment for preventing recurrence of digital ulcers.
- **Endothelin receptor antagonist**
  The preventive efficacy of bosentan on the appearance of new ulcers has been demonstrated in two controlled trials. This efficacy is more pronounced in the higher-risk forms of multiple digital ulcers (**use within the European MA**). The drug is administered at a dosage of 62.5 mg twice per day for 4 weeks, then at 125 mg twice per day. The optimal duration of use (occasional treatment especially in cold weather or continuous treatment) remains to be defined. Effective contraceptive method in childbearing age is needed.

#### Cutaneous sclerosis treatment

There is no consensus on the use of low dosages of prednisone (≤ 15 mg/day) in edematous cutaneous SSc. The use of corticosteroids at low doses in this indication requires a good evaluation of the risk-to-benefit ratio, including the risk of precipitating a scleroderma renal crisis, although usually this risk is reported for dosages > 15 mg/day (**off-label use**).

- Methotrexate

Two small studies have shown a moderate benefit of methotrexate (MTX) on skin damage. An analysis of the last placebo-controlled trial using a Bayesian method suggested that the probability of MTX improving the mRSS was 94%. The EUSTAR recommendations are in favor of using MTX in recent diffuse forms of SSc. The working group includes MTX among the possible therapeutic options for diffuse forms of SSc. The recommended dosage must not exceed 0.3 mg/kg per week taken orally or subcutaneously. There is no established treatment duration, but if there is clinical improvement, the working group recommends a treatment duration of at least 2 years (**use within MA**).

- Cyclophosphamide

There is no available trial evaluating the efficacy of cyclophosphamide as DMARD for SSc. On the other hand, there are trials that evaluated this drug against placebo for ILD. In the SLS-I study, oral cyclophosphamide produced a 3.6-point reduction in Rodnan score, which was significant after 12 months of evaluation. This effect disappeared 1 year after stopping the cyclophosphamide. In a European observational trial of the treatment of recent diffuse forms of SSc, cyclophosphamide use was associated with a 3.3 drop in the Rodnan score after 12 months, which did not differ significantly from the drop observed in patients untreated by immunosuppressants (**off-label use**).

- Mycophenolate mofetil

There is no direct study of the use of MMF as DMARD of SSc, but there are observational studies and also controlled studies for lung damage. In the SLS-II trial, MMF use was associated with a 4.9-point reduction in the modified Rodnan score versus 5.3 for cyclophosphamide at 24 months. The results are only significant in diffuse forms of SSc. Analysis of SLS-II compared with the SLS-I placebo group would suggest that MMF use was associated with an improvement of the modified Rodnan score compared with the placebo group after 24 months. This result coincides with several observational studies and literature reviews that suggest an effect of MMF on the skin damage from diffuse forms of SSc. In light of these data, the working group considers MMF a DMARD to be considered in the diffuse cutaneous forms of SSc with or without lung damage. The recommended dosage of MMF is from 2 to 3 g/day in direct treatment of ILD (**use within MA**).

In initially severe cutaneous forms, cyclophosphamide, MMF, or autologous hematopoietic stem cell transplants (see Sect. 5.5.10, cellular therapies) are to be considered from the start (to be discussed at the MCM).

The skin must be treated locally for good hydration. It is recommended to use moisturizing and softening creams and lotions several times a day. Paraffin baths for the hands or the use of castor oil have not been rigorously studied scientifically. Personalized physiotherapy with massages aimed at softening the skin or subcutaneous tissues can be proposed, although no rigorous study on the subject has been conducted to date (working group opinion).

In case of pruritus, an H1-antihistimine can be proposed, carefully avoiding any interaction with other drugs that could lengthen the QT, especially domperidone and metoclopramide. Additional treatment may include topical emollients, increasing hydration, and UV therapy.

#### Treatment of locomotive apparatus damage

Articular and periarticular damage

Locomotive apparatus damage is frequent with SSc: arthralgias, arthritides, and tenosynovitis are especially common in the first years of the disease. Fibrous tenosynovitis is included in the disease activity score and is considered a sign of worsening development. No randomized study has specifically addressed musculoskeletal damage.

- Arthralgias and arthritis can be treated by analgesics and by nonsteroidal antiinflammatory drugs (NSAIDs) in the short term, with monitoring of renal function and after evaluation of digestive bleeding risk.
- Oral corticosteroids are commonly proposed at an initial dosage of prednisone equivalent not exceeding 10 to 15 mg/day, then at a lower long-term dosage of less than 10 mg/day.
- Corticosteroid shots can be proposed in case of articular or tenosynovial damage, and also carpal tunnel syndrome.
- Tenosynovitis can benefit from the same treatments as articular damage, but there has not been any specific trial dedicated to this type of damage.
- Rehabilitation programs can reduce disability, but they have not been shown to have significant long-term efficacy.

No **csDMARD** (conventional synthetic disease-modifying antirheumatic drug) has been specifically evaluated in a controlled randomized trial for joint damage from SSc. However, expert recommendations have been published.

- Methotrexate: it showed improvement of skin damage and quality of life in a randomized trial, but articular damage was not measured. However, it is often proposed in cases of polyarticular involvement with an inflammatory component, by analogy with rheumatoid arthritis, especially in early diffuse cutaneous forms. It is also used in the erosive forms. Subcutaneous administration can be used, especially in case of gastrointestinal damage (**off-label use**).
- Oral cyclophosphamide has not been shown to be effective on joint damage.

**Certain bDMARDs** (biologic DMARDs) have been the object of observational studies and other randomized therapeutic trials (**off-label use**):

- Rituximab, an anti-CD20 antibody, has marketing authorization for rheumatoid arthritis and can be discussed in case of refractory arthritis, particularly in case of overlap with rheumatoid arthritis.
- Tocilizumab, an anti-IL6 antibody, has marketing authorization for rheumatoid arthritis and can be discussed in case of refractory arthritis, particularly in case of overlap with rheumatoid arthritis.
- Abatacept (CTLA4-Ig) has been evaluated in a phase 2 trial. The result was negative for its main criterion (Rodnan score). This treatment cannot be recommended for this indication except in case of overlap with rheumatoid arthritis.
- Anti-TNFα agents have no clearly demonstrated efficacy, and progression of interstitial lung disease is feared in the event of preexisting pulmonary involvement. In this context, use of anti-TNFα agents is not recommended by the working group.

The working group recommends the use of biotherapy only in case of insufficient efficacy of methotrexate combined with corticosteroid therapy not exceeding 10 to 15 mg/day of prednisone in the attack phase.

- Muscular damage

Inflammatory muscular involvement authenticated by imaging, and ideally histologically, sometimes justifies recourse to high-dose corticosteroid therapy. Such a decision will only be taken in case of symptomatic involvement and in case of inflammatory infiltrates present in the muscular biopsy. The risk of scleroderma renal crisis, especially in patients with recent diffuse cutaneous SSc, leads in this case to prescribe corticosteroids at a dosage not exceeding 0.5 mg/kg/day in combination with methotrexate and possibly with IVIG at a dose of 2 g/kg in monthly treatment if renal function allows it, in severe or refractory forms. An appropriate and personalized functional program of rehabilitation can be proposed. Moderate paucisymptomatic forms (which are the most frequent) do not require specific treatment.

Supervised rehabilitation programs allow for reducing the disability in the short term. However, the effectiveness of rehabilitation in the longer term depends on patient adherence to the program.

Fibromyalgia can be associated with musculoskeletal manifestations of SSc and must be identified semiologically and clinically. Programs of specific treatment can then be proposed.

- Calcinoses

The frequency of this complication is high (near 25% of all patients) and is the source of pain and disability. No treatment has been shown to be effective for this complication.

Colchicine is often proposed in case of inflammatory flare-ups (**off-label use**).

Vitamin K antagonists and bisphosphonates should not be used for this indication.

In certain situations, surgical excision of calcium deposits can be proposed in order to promote healing and avoid secondary infections after making sure that the peripheral vascular condition allows for it.

Intravenous sodium thiosulfate has no indication in this type of calcinosis. Topical application is proposed by some teams for inflammatory forms. And finally, intralesional injections are still under investigation (**off-label use**).

- Bone damage

There has not been any study on osteoporosis treatment for SSc.

- Preventive hygieno-dietary measures for osteoporosis are indispensable. Calcium supplements, along with the correction of vitamin 25-OH-D3 deficiency, must be systematically screened for and corrected.
- Specific drugs (anti-resorbers or anabolics) are to be discussed with the corresponding rheumatologist in the case of proven osteoporosis. In case of digestive damage, intravenous forms of bisphosphonates must be preferred due to the elevated risk of malabsorption. Subcutaneous denosumab every 6 months is to be discussed in case of proven osteoporosis with high risk of fracture (see HAS recommendations:
- https://www.has-sante.fr/portail/jcms/c_1194578/fr/osteoporose).

#### Interstitial lung disease treatment

Before starting treatment of ILD, it is necessary to evaluate its severity by evaluation of dyspnea, a 6-min walk test, percutaneous oxygen saturation, FVC and TLC, DLCO, and data from a high-resolution CT scan of the chest (extent of lesions in % of lung affected and their characteristics: ground glass/honeycomb/bronchiectases). Specific treatment depends on the progressive character (or not) of the ILD, defined by the loss of at least 10% of the FVC in absolute or relative terms over 12–18 months or the loss of at least 15% of the DLCO in absolute or relative terms. These criteria are validated for idiopathic ILD but not for SSc, the degradation of respiratory function being much slower in this disease. In this context, the progression of ILD evaluated over the past 24 months could be based on: (1) a drop in FVC of at least 10% of the predicted value; (2) a drop in FVC of 5–10% of the predicted value WITH increased respiratory symptoms OR extension of the fibrosis on high-resolution CT scan of the chest; (3) increased respiratory symptoms WITH extension of the fibrosis on high-resolution CT scan of the chest.

The duration of the SSc’s progression, the extension and type of CT scan lesions, as well as dyspnea will also be taken into account. The Goh classification can be used to evaluate the severity of ILD: limited ILD if the extent of the CT anomalies is less than 20% of the pulmonary surface (or FVC ≥ 70% when the CT extent is undetermined); extended ILD or FVC < 70% if the extent of CT abnormalities exceeds 20% of the pulmonary surface.

Whatever the criteria used, before considering a specific treatment for ILD associated with SSc, it is important to look for certain damage which can impair the respiratory function of patients and which does not fall under the specific treatment of fibrosis (pulmonary embolism, severe anemia, etc.); specific situations affecting the prognosis of ILD associated with SSc:

- Morbid obesity, which would be a source of overestimation of the loss of pulmonary volumes.
- Association with obstructive sleep apnea syndrome: polysomnography could be conducted, especially in case of extensive ILD.
- An emphysema and fibrosis syndrome (existence of emphysema associated with the ILD) which can underestimate the restrictive syndrome associated with the ILD. These patients generally have a lower DLCO and higher need for oxygen.
- Pulmonary hypertension (PH) associated with ILD (group 3 PH): it can be suspected by echocardiogram, especially if the estimated SPAP is ≥ 40 mmHg. At times this pulmonary hypertension can be severe. Only right heart catheterization will allow the evaluation of the hemodynamic severity of this pulmonary hypertension. Its indication is to be discussed on a case-by-case basis at the multidisciplinary coordination meeting.

Symptomatic treatments

- Total and definitive smoking cessation and avoidance of secondary smoke;
- Vaccinations: it is recommended to give the annual flu vaccination and the anti-pneumococcal vaccination (13-valent vaccine followed at least 2 months later by the 23-valent vaccine) to all patients with confirmed ILD. The anti-Haemophilus influenzae vaccine is not included in a specific recommendation but is left to the choice of the prescriber. It is however recommended in case of hyposplenia or splenectomy;
- Optimization of gastroesophageal reflux treatment: GERD, nearly constant in SSc, is a potentially aggravating factor of ILD. In case of GERD symptomatology or esophageal stasis, the association of PPI and hygieno-dietary measures is required.
- Oxygen therapy: as with other causes of chronic respiratory failure, long-term oxygen therapy is recommended in the event of severe respiratory failure defined by PaO_2_ ≤ 55 mmHg (7.3 kPa), or PaO_2_ between 55 and 60 mmHg (7.3–8.0 kPa) with at least one of the following criteria: polycythemia (hematocrit > 55%), signs of pulmonary hypertension, signs of right heart failure;
- Respiratory rehabilitation: a respiratory rehabilitation program must be discussed on a case-by-case basis for all patients with exertional dyspnea;
- Treatment of the cough: there is no precise recommendation concerning treatment of the cough associated with ILD. The first approach is to ensure that it is not linked to GERD. There is no study concerning inhaled corticosteroids (**off-label use**), but they can be tested on a case-by-case basis;
- Nutritional state: the nutritional state of each patient must be assessed and any malnutrition corrected in order to improve anti-infectious defenses.

**DMARDs**

Due to a favorable expert consensus, the working group recommends treating patients with progression (loss of 10% of FVC or ≥ 200 ml and/or 15% of DLCO or ≥ 3 ml/min/mmHg in the past year) or patients with severe cases from the start.

- The use of intravenous cyclophosphamide is based on a French retrospective study on scleroderma patients with aggravated ILD. The schedule of administration every 4 weeks is that usually used for ILD from connective tissue diseases. The dose is 0.7 g/m^2^ or 0.5 g/m^2^ in patients over 65 years of age or with GFR < 30 ml/min/m^2^, at the rate of one treatment every 28 days for 12 months. The dose of cyclophosphamide is capped at 1200 mg/injection. Intravenous uromitexan is administered concomitantly dose for dose. The prevention of *Pneumocystis jiroveci* infections must be routine and is based on prescribing trimethoprim 80 mg/day + sulfamethoxazole 400 mg/day (or trimethoprim 160 mg + sulfamethoxazole 800 mg three times per week). In case of sulfonamide allergy, pentamidine aerosol (300 mg/dose) every 3 or 4 weeks or atovaquone orally (1500 mg/day) can be given. The total duration of one year of treatment is justified by the fact that, after a 6-month treatment of intravenous cyclophosphamide followed up with PO azathioprine for 18 months, some initially responsive patients worsened afterwards.
- MMF at a dose of 1500 mg, twice a day for 2 years has shown noninferiority in a randomized study carried out versus oral cyclophosphamide (**off-label use**) and may be an alternative as a first-line treatment, especially for forms of ILD with poorer prognosis, even if they do not meet the usual criteria for progression (i.e., loss of at least 10% FVC or 15% DLCO over a period of 12 months). In this study, the patients had SSc with a course of less than 7 years from the first non-Raynaud symptom, ILD from high-resolution CT scan, and on PFT had FVC between 45% and 79%, DLCO ≥ 40%, and dyspnea of at least NYHA class II.
- Despite the absence of a controlled study, the working group recommends maintaining immunosuppressant treatment (azathioprine 1–2 mg/kg/day or MMF 1 g × 2/day) (**off-label use**) as a relay for the cyclophosphamide if it was chosen as first-line treatment. In patients with cyclophosphamide failure or new progression under azathioprine maintenance treatment, MMF can be proposed, although it has not been shown to be superior (**off-label use**).
- Low-dose corticosteroid doses are recommended by some experts in association with cyclophosphamide or MMF. Given the risks of a renal crisis occurring in scleroderma patients, we recommend the use of corticosteroids at dosages ≤ 15 mg/day of oral prednisone (**off-label use**).
- RTX was studied in a small randomized trial with positive results for the main criterion (FVC). Several retrospective studies have shown discordant results, some positive, others not, regarding the use of RTX, with one European study in particular finding no improvement of ILD from RTX. Because of this, in patients with treatment failure from cyclophosphamide and/or MMF, use of rituximab must be discussed in a MCM (**off-label use**).
- Tocilizumab has been assessed in recent cutaneous forms with inflammatory profile. Both the phase 2 and phase 3 studies were negative for the primary criterion (Rodnan score). For one of the secondary criteria (pulmonary interstitial damage), there was a definite preservation of lung volumes (FVC). There has not yet been a study conducted on patients with established ILD. The use of tocilizumab in this indication can only be considered after validation in a MCM (**off-label use**).
- Nintedanib is a tyrosine kinase inhibitor involved in angiogenesis and fibrosis. A phase 3 trial showed that, at the 150 mg dose morning and evening, it was able to slow down the degradation of FVC in patients with ILD affecting at least 10% of the pulmonary parenchyma. The therapeutic benefit is statistically significant but modest (delta FVC = 41.0 ml/year). Nintedanib can be associated with immunosuppressants, especially MMF. Another trial has just confirmed a benefit on the loss of FVC (delta FVC = 107.0 ml/year), in a larger group of patients with active fibrotic interstitial disease. Its frequent side effects (diarrhea, disturbances of hepatic balance) can be a limiting factor, but can also be managed, for the diarrhea, by symptomatic treatments. Nintedanib is currently only approved for idiopathic pulmonary fibrosis and for SSc-associated ILD..
- Therapeutic intensification with autologous hematopoietic stem cell transplant can be discussed and can be considered in certain well-selected patients with a rapidly progressive form of SSc (see Appendix 4) and must be performed at expert centers accredited for these procedures.
- Lung transplant: in the forms of ILD with severe respiratory failure in spite of the treatments previously mentioned, and in the absence of other severe visceral damage, a lung transplant can be considered. The results of a pulmonary graft are superimposable in terms of survival to those obtained for patients having received a lung transplant for an idiopathic pulmonary fibrosis. A French group of experts has established a list of absolute or relative contraindications for lung(/heart) transplant in patients with SSc (Appendix 5).

In practice, it appears to the working group that the choice for recent ILD from SSc would now be MMF as first-line treatment, and intravenous cyclophosphamide as second-line or as first-line in rapidly progressing forms, or forms with poor prognosis (extended form according to the Goh classification) and rituximab as third-line treatment. The place of nintedanib still needs to be clarified, but it must undoubtedly be reserved for confirmed forms. It is recommended to restrict its indication to progressive forms. In early and severe forms, a combination of MMF + nintedanib could be eventually considered (to be discussed in MCM).

Every therapeutic line must be assessed after 6 months of treatment based on clinical data (NYHA functional class, 6-min walk test) and PFT. A thoracic high-resolution CT scan should be conducted at the end of a therapeutic sequence and in case of clinical aggravation or PFT. In case of clinical/PFT/CT scan stabilization or improvement, immunosuppressant therapy should be continued for at least 2 years and probably longer (although published data are lacking). Intravenous cyclophosphamide can be continued for 12 months and will then relay with azathioprine or MMF.

#### Treatment of pulmonary arterial hypertension (PAH)

The recommendations for management of patients with PAH associated with SSc are based on those for idiopathic PAH according to the joint recommendations of the European Societies of Cardiology and Pulmonology.

Any patient with SSc suspected of pulmonary hypertension requires a multidisciplinary coordination meeting in order to establish the indication of right cardiac catheterization, the only examination allowing confirmation of the diagnosis of pulmonary hypertension (mean PAP ≥ 20 mmHg with occluded PAP ≤ 15 mmHg and PVR ≥ 3WU). Any patient diagnosed with pulmonary hypertension must be presented to the reference center or to a competence center for pulmonary hypertension. Several pathologies can lead to pulmonary hypertension with SSc:

- PAH (Group 1.4)
- PAH with veno-occlusive component (group 1.6)
- PH in connection with left cardiopathy (group 2)
- PH in connection with a pulmonary pathology and/or hypoxemia (group 3)
- Chronic thromboembolic PH (group 4)

When the diagnosis of group 1 pulmonary hypertension has been established, therapeutic management is based on the following strategy:
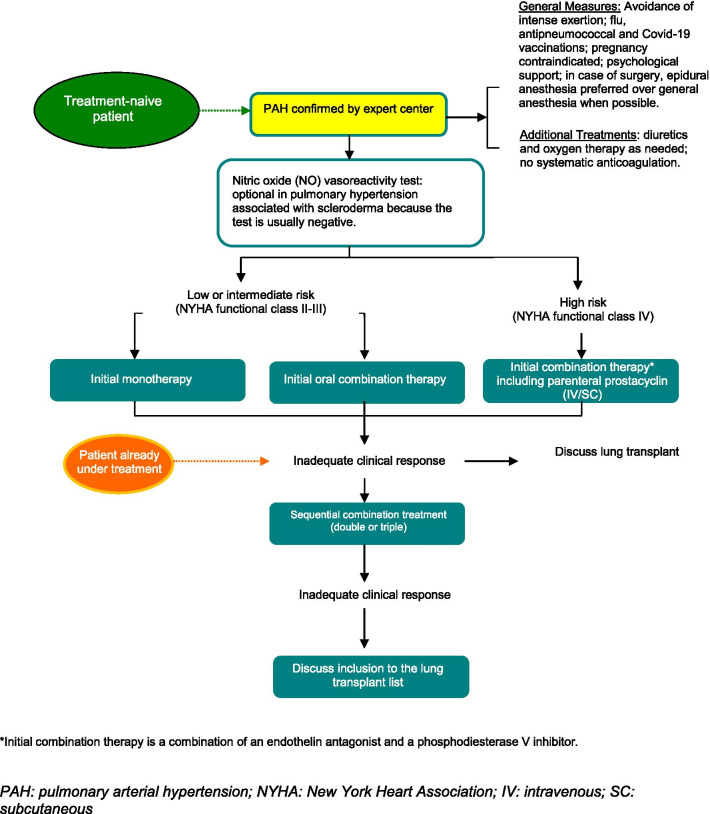


Nonspecific treatments

- **Anticoagulant and diuretic treatment**

- Two registry studies have not shown any benefit from anticoagulant therapy in terms of survival in PAH associated with SSc. Instead, it would even be harmful because of the increased risk of digestive hemorrhage in patients with gastrointestinal telangiectasias. The working group does not recommend their use for this indication outside of special situations such as the presence of an indwelling catheter for continuous injection of epoprostenol or the existence of an associated authentic antiphospholipid syndrome (or the existence of another indication such as atrial fibrillation). For the moment it is advised to use vitamin K antagonists rather than direct oral anticoagulants. The risk-to-benefit ratio should always be defined before starting anticoagulant treatment.
- Diuretic therapy associated with a salt-free diet should be combined with specific treatments if there are signs of right heart overload.

(b) Oxygen therapy

- This is most frequently prescribed when there is significant hypoxemia (PaO_2_ < 60 mmHg), primarily for the symptoms.
- Its benefit in case of true shunt or serious desaturation with exertion is questionable.

(c) Flu, pneumococcal, and COVID-19 vaccinations are recommended.
(d) Therapeutic patient education

The occurrence of PAH in a patient with SSc warrants referral to a specific PAH program.

- Specific treatments

The treatments below are proposed in case of PAH after coordination with a reference or competence center for pulmonary hypertension. They have no indication in the other forms of pulmonary hypertension which can be observed in SSc. They are validated in patients of group 1.4 with MPAP ≥ 25 mmHg and PVR ≥ 3 WU. Targeted studies on patients with MPAP between 21 and 24 mmHg are needed before extending the indications.

One particular form of PAH, involving pulmonary venules (veno-occlusive disease), is frequently observed in SSc. Acute pulmonary edemas can be observed at the start of these treatments (especially in case of the use of prostacyclin analogs or derivatives) in these forms. Recourse to these treatments must be done in a specialized setting in case of suspected veno-occlusive disease.

a.** Medicinal**

**Calcium channel blockers**

There is no indication for calcium channel blockers in the treatment of SSc-associated PAH because patients are most frequently acutely nonreactive to nitric oxide.

As many patients are treated with calcium channel blockers for their Raynaud's phenomenon or digital ischemic disorders, these can be maintained in case of PAH even in long-term nonresponding subjects provided that they are used at low dosage, and provided that a nonbradycardic calcium channel blocker is chosen that will not worsen the functional symptoms (thus amlodipine or nifedipine rather than diltiazem or verapamil).

**Endothelin receptor antagonists (use within MA)**

Bosentan is an orally active mixed ETA and ETB receptor antagonist approved for PAH associated with a connective tissue disease of NYHA functional class II or III. Bosentan is started at a dosage of 62.5 mg mornings and evenings for 4 weeks, then increased to a dosage of 125 mg mornings and evenings according to hepatic tolerance (monthly hepatic testing obligatory (SGOT, SGPT) and routine monitoring of hemoglobin).

The benefit also provided by bosentan on the secondary prevention of digital ulcers can lead to recommending first-line use of bosentan in case of SSc-associated PAH if the patient has a severe digital ulcer disease.

Ambrisentan is an orally active ETA receptor antagonist approved for PAH associated with connective tissue disease of NYHA functional class II or III. The dosage is 5 mg once per day and can be increased to 10 mg per day. Monthly hepatic testing is recommended but not mandatory. A complete blood count is also recommended every 1–3 months. Ambrisentan is contraindicated in cases of idiopathic pulmonary fibrosis, but there are no data concerning SSc-associated ILD. The working group does not recommend its use in case of associated extensive ILD.

*Phosphodiesterase 5 inhibitors*: oral sildenafil and tadalafil (use within MA)

Sildenafil and tadalafil are approved in the treatment of idiopathic, familial, or SSc-associated PAH with dyspnea of NYHA functional classes II or III. The dosage used is 20 mg three times per day for sildenafil and 2 × 20 mg once for tadalafil. There is no specific biological monitoring for these treatments.

**Soluble guanylate cyclase stimulators (off-label use)**

Oral riociguat received European authorization for the treatment of connective tissue disease-associated PAH, but is only reimbursed in France in the context of chronic thromboembolic PH. It is contraindicated in cases of idiopathic pulmonary fibrosis.

**Continuous intravenous prostacyclin injection (epoprostenol) (use within MA)**

Epoprostenol is approved in the treatment of PAH associated with connective tissue disease of NYHA functional class III or IV. It is administered in continuous intravenous perfusion through a portable perfusor connected to a tunneled central venous catheter. In urgent situations, it can be administered through a peripheral venous route for short duration while the central venous route is being set up. Epoprostenol constitutes the reference treatment for severe forms of SSc-associated PAH of functional classes III/IV.

**Prostacyclin analogs (off-label use)**

- Iloprost in aerosol form is approved in the treatment of familial idiopathic PAH for patients in NYHA functional class III. There is **no MA for SSc.** In practice, it is not recommended to use iloprost aerosols as a first-line treatment in SSc-associated PAH because of the existence of possible therapeutic alternatives and the rebound effect risk. The benefit of this association with other drugs such as sildenafil or endothelin receptor antagonists can be discussed on a case-by-case basis with a reference or competence center.
- Treprostinil administered by continuous subcutaneous injection is approved in the treatment of familial or idiopathic PAH for NYHA functional class III. There is **no MA for SSc.** According to the Pulmonary Vascular Disease Working Group of the Société de Pneumologie de Langue Française [Francophone Pneumology Society], SSc-associated PAH is one of the relevant indications of treprostinil. Its use with SSc can be hindered in cutaneous forms with abdominal skin damage, because the injections are usually done in the abdomen.

**Prostacyclin receptor agonists (use within MA)**

Selexipag is a selective prostacyclin receptor agonist that is used orally. It is approved for treating PAH associated with connective tissue diseases of NYHA functional class III and insufficiently controlled by treatment associating an endothelin receptor antagonist and a phosphodiesterase 5 inhibitor. It is prescribed at a progressive dose over several weeks up to the maximum tolerated dose, to a maximum of 1600 μg 2 times a day, based on tolerance. Special monitoring for the occurrence of adverse effects is indispensable (headaches, flushing, digestive disorders, etc.).

b.** Surgery**

*Atrial septostomy*

This is a therapeutic alternative that can be useful for severe PAH, in particular in patients on the lung transplant list whose condition continues to deteriorate in spite of maximum medical treatment. However, it is not routinely done in France.

*Lung or heart–lung transplant*

This is the last recourse in case of severe PAH insufficiently improved by maximum medical treatment. The indication of transplant is systematically posed by reference centers or a competence center.

There are SSc-specific contraindications to lung transplant (see Appendix 5).

For more information on the therapeutic strategy for PAH, refer to the PAH NDCP (http://www.has-sante.fr). Regardless of the first-line treatment chosen (monotherapy or combination treatment), patients must be re-evaluated every 3 or 4 months. Absence of improvement in functional class, absence of significant improvement in the 6-min walk test and/or hemodynamic parameters, appearance of signs of right heart failure, or aggravation of PAH symptoms should be considered indicators of therapeutic failure. A new right heart catheterization should be discussed in partnership with the reference center or a competence center before considering more intense therapies. An initial combination treatment should be discussed with the reference or competence center. A follow-up right heart catheterization is recommended 4–6 months after the initial treatment or after therapeutic intensification. The absence of improvement should lead to quickly discussing oral bi- or tri-therapy or the use of injectable prostacyclin analogs.

When the diagnosis of group 1 pulmonary hypertension has been established (PAH), the therapeutic management is based on the following strategy:

#### Treatment of heart diseases

All heart tunics can be affected: endocardial, myocardial, and pericardial, possibly causing atrial and/or ventricular rhythm disorders, conduction disorders, myocardial ischemia, pericardial effusion, and heart failure.

We should also add the possible existence of pulmonary hypertension (PH) related to this left heart disease (post-capillary PH, from group 2). This form of PH can coexist with a PH of other mechanisms in the same patient (see forms of PH in SSc). In addition, during the evolution of SSc, some PHs may change category or combine.

The prevalence of atherosclerosis does not appear to increase during SSc.

Heart disease has a poor prognosis, and its management depends on its detection and proper management.

•** Pericarditis**

In case of symptomatic pericarditis, treatment with NSAIDs (use with caution in case of upper digestive problems)/colchicine may be offered as a first line of treatment. Rare compressions may justify high doses of corticosteroids in combination with pericardial drainage.

• ** Therapeutic management of arrhythmia and conduction disorders**

Arrhythmia must be treated with the usual anti-arrhythmics with the usual precautions (no class 1 anti-arrhythmics in case of ischemic heart disease and/or left ventricular dysfunction, check for absence of QT prolongation, etc.). Beta-blockers are not contraindicated, but their use is limited due to the risk of aggravation of Raynaud's phenomenon and digital ulceration, with a preference for cardioselective blockers. Beta-blockers and amiodarone may promote development of pulmonary fibrosis.

Anticoagulant treatment is necessary for supraventricular rhythm disorders unless the CHA2DS2-VASc is 0 (http://www.cardiologie-francophone.com/PDF/scores/score-CHA2DS2-VASc%20.pdf). If the score is 1, oral anticoagulant therapy with a VKA (INR 2–3) or a direct thrombin inhibitor (dabigatran) or an oral factor Xa inhibitor (rivaroxaban, apixaban) should be considered, based on a risk assessment for bleeding and patient preference.

A significant conduction disorder will require the insertion of a pacemaker.

Expert advice is required to determine whether there is an indication for the insertion of a defibrillator.

• ** Myocardial disease**

Table 7 summarizes the major cardiac manifestations that may occur during the course of SSc and their principles of management. A calcium channel blocker treatment may be offered to improve perfusion and coronary reserve. Angiotensin-converting enzyme (ACE) II inhibitors can replace them in cases of intolerance, or be added, particularly when there is patent ventricular dysfunction. In advanced dysfunction, conventional treatment of systolic heart failure should be offered in the absence of contraindications (ACE inhibitors or angiotensin receptor blockers II (ARB II) and beta-blockers at maximum tolerable doses, mineralocorticoid receptor antagonists, diuretics at minimum doses to control congestion, ivabradine in case of sinus tachycardia > 70/min, possibly digoxin). In advanced forms (left ventricular ejection fraction ≤ 35%), the possibility of a defibrillator, resynchronizer (in case of wide left branch block), or replacement of ACE/ARB II inhibitors by mixed angiotensin II and neprilysin receptor inhibitors (ANRI, sacubitril-valsartan) should be discussed. For heart failure with preserved ejection fraction, the therapy is symptomatic, mainly based on diuretics. Very rare myocarditis may justify the use of immunosuppressants, but no precise recommendations exist in this area (just expert opinion).

**Table 7:** Possible causes of cardiac symptoms with a suggestion for further exploration and therapeutic management

Disease type/symptom	Order of prevalence	Examinations to be discussed on a case-by-case basis	Treatment proposals to be discussed on a case-by-case basis
**Coronary** **microcirculatory disorder**	> 60%	EKG Troponin Myocardial scan* Cardiac MRI	Calcium channel blocker ACE/ARB II Control of risk factors
**Coronary macrocirculatory disorder**	< 10%	EKG Troponin Myocardial scan* Cardiac stress MRI Stress echocardiography Coronary scan Coronary angiography	Control of risk factors Treatment based on recommendations Revascularization
**Systolic heart failure** **If LVEF ≤ 40%**	5%	EKG Echocardiogram Natriuretic peptides Troponin Myocardial MRI	Based on recommendations: ACEi or ARB II (ANRI, sacubitril, valsartan in second line)/cardioselective beta-blocker to be discussed according to ILD and Raynaud/digital ulcers Mineralocorticoid receptor antagonists Defibrillator…
**Preserved LVEF heart failure**	30–35%	EKG Echocardiogram Natriuretic peptides Troponin Cardiac MRI	Diuretics
**Isolated VD dysfunction**	38%	EKG Echocardiogram Natriuretic peptides Troponin Cardiac MRI Right heart catheterization	Diuretics
**Pericardial effusion**	Frequent	Echocardiogram Cardiac MRI	Usually monitoring Colchicine+++
**Constriction**	Rare	Echocardiogram Cardiac MRI Right heart catheterization	Diuretics, lower doses if bradycardia medication Pericardiectomy
**Pulmonary hypertension Precapillary**	< 15%	EKG Echocardiogram (VTI) Natriuretic peptides Troponin Right heart catheterization	Specific treatment of PAH if group 1 Treatment of ILD if group 3
**Pulmonary arterial hypertension** **With veno-occlusive component**	Rare	Right heart catheterization Chest scan	Diuretics Specific treatment of PAH
**Pulmonary hypertension Post-capillary**	< 12%	EKG Echocardiogram Natriuretic peptides Troponin Cardiac MRI Right heart catheterization	Treatment of heart failure
**Tachycardia** **Supraventricular**	15%	EKG Holter monitor	Anti-arrhythmic treatment based on recommendations Anticoagulant treatment
**Tachycardia Ventricular**	Rare	EKG Holter monitor Echocardiogram Look for myocardial ischemia	Antiarrhythmic treatment based on recommendations Defibrillator Treatment of ischemia
**Bradycardia and conduction disorders**	Rare	EKG Holter	Stop bradycardia medication Stimulator

^*^Myocardial MRI is still being evaluated for the study of microcirculation; myocardial scintigraphy is still more suitable now

*NSAIDs* nonsteroidal antiinflammatory drugs, *ANRI* angiotensin II receptor neprilysin inhibitor, *ARB II* angiotensin II receptor blockers, *LVEF* left ventricular ejection fraction, *PAH* pulmonary arterial hypertension, *ACE* angiotensin-converting enzyme inhibitor, *MRI* magnetic resonance imaging, *ILD* diffuse infiltrating lung disease, *VTI* rate of tricuspid insufficiency

The working group recommends immunosuppressive therapy for symptomatic heart disease with MRI-confirmed myocarditis.

#### Treatment of scleroderma renal crisis

• Preventive treatment

Prophylactic administration of ACE inhibitors has not yet been shown to be effective in preventing the occurrence of CRS. In contrast, treatment with prednisone at a dosage > 15 mg/day within the previous 3 months appears to be associated with the occurrence of CRS (Table 2).

In this context, the prescription of corticosteroids should always be subject to expert advice.

At-risk patients should receive therapeutic education and perform regular blood pressure monitoring by self-measurement of their blood pressure according to the rule of 3.

• Curative treatment

**Fig. 4:**
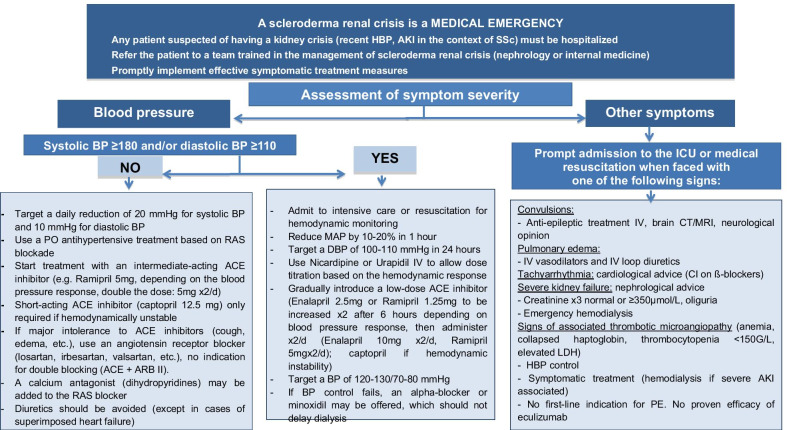
Management of scleroderma renal crisis. *BP* blood pressure, *PO* per os, *RAS* renin–angiotensin system, *ACE* conversion enzyme inhibitors, *ARB II* angiotensin receptor blockers II, *MAP* mean arterial pressure, *IV* intravenous, *CT* computerized tomography, *MRI* magnetic resonance imaging, *CI* contraindication, *IV* intravenous, *HBP* high blood pressure, *AKI* acute kidney injury, *PE* plasma exchange, *LDH* lactate dehydrogenase

The main issue is early control of blood pressure with a goal of ≤ 130–120 mmHg for systolic blood pressure and < 80–70 mmHg for diastolic blood pressure within 72 h after starting treatment. A standard treatment regimen is proposed (Fig. 4). Currently, there is no demonstrated indication for first-line plasma exchange, eculizumab, or immunosuppressants. Corticosteroid therapy is contraindicated.

a. Antihypertensive treatment

A proposal for management based on symptom severity is summarized in Fig. 4.

- ACE inhibitors: the only therapeutic class to have demonstrated efficacy and to have modified the CRS prognosis. This demonstration is based on cohort follow-up studies. In the absence of hemodynamic instability, we recommend the use of an intermediate half-life ACE inhibitor such as enalapril or ramipril in graduated doses. Captopril (short-acting ACE inhibitor) is only used in cases of hemodynamic instability. Conventional increases in creatinine levels with ACE inhibitors (lowering of renal perfusion pressure) should not result in a decrease in dosage.
- Nicardipine (Loxen) or urapidil (Eupressyl) can be used early if blood pressure is not controlled by ACE inhibitors alone or in cases of “malignant HBP” in order to titrate the dosage and allow a controlled drop in blood pressure while preserving cerebral circulation. The use of iloprost is recommended by some authors, but not validated (**off-label use**). Use of bosentan cannot currently be recommended in the absence of clinical studies demonstrating its efficacy.
- Vascular filling should be considered in patients without heart failure but with a malignant HBP pattern and evidence of hemoconcentration.

b. Extrarenal purification

The use of extrarenal purification should be considered early if renal function is rapidly deteriorating. Pathway problems in these patients should not delay its implementation.

c. Treatment of normotensive forms

- In these forms, systolic BP may remain “abnormally high” (130–150 mmHg) and the use of low-dose ACE inhibitors initially is recommended.
- The use of a low-dose, medium half-life ACE inhibitor should be considered (such as ramipril 1.25 mg × 2/day) with gradual titration of the increase (up to 5 mg × 2/day) under control of the hemodynamic tolerance (systolic BP targets ideally maintained between 110 and 120 mmHg).
- Systolic BP should be maintained ≥ 100–110 mmHg to avoid the formation of post-ischemic tubular necrosis lesions (especially in elderly patients).
- If the systolic BP falls too much (< 100 mmHg), the next dose should not be given and the dosage should be reduced on resumption. Isotonic saline may also be administered to restore the systolic BP to an acceptable level (> 110 mmHg).
- BP monitoring during this adaptation phase is recommended.

a.** Evolution in dialysis and transplant**

- Withdrawal from dialysis may be observed within the first 2 years after the occurrence of CRS in patients maintained on ACE inhibitors.
- Therefore, it is commonly accepted that scleroderma patients should not be transplanted until after this 2-year period. At 5 years, patient survival is 82% and transplant survival is 93% (after censoring for deaths).

#### Treatment of digestive disorders

• Esophagitis and esophageal motor disorders

The treatment of gastroesophageal reflux disease and its complications is based on:

- Hygieno-dietary measures, such as reduced-sized meals, reducing or even stopping consumption of tobacco, alcohol, tea, coffee, and chocolate. Consultation with the nutrition team or a dietician is recommended. TPE sessions are a good complement to the patient’s management of their digestive disorders.
- Postural rules, namely raising the head of the bed and avoidance of decubitus for 3 h after meals.
- Anti-secretory therapy: double- and even quadruple-dose proton pump inhibitors.
- Prokinetics: metoclopramide and domperidone are to be discussed, but with caution and minimal dosage, with close monitoring of the risk of adverse effects of these treatments (risk of QT prolongation for domperidone). In any case, they should be taken at least 30 min before a meal. For domperidone, in 2014, following European recommendations aimed at minimizing cardiac risks, the ANSM informed healthcare professionals to prescribe domperidone at the lowest possible effective dose and for the shortest possible treatment duration, generally not exceeding 1 week. However, some patients do benefit from it over the long term.
- The management of gastroparesis (see below) and constipation, which can be aggravating factors.
- Endoscopic dilatations are sometimes necessary for peptic stenoses that are resistant to medical treatment. The working group does not recommend antireflux surgery, which has no proven efficacy.

• Gastroparesis (delayed abdominal pain and vomiting)

Dietary management is always necessary (fragmentation of meals, mixed diet). Low-residue diets and vitamin supplements have been recommended based on empirical evidence.

Stomach prokinetics accelerate gastric emptying, but can have a negative effect on motility of the small intestinal when prescribed at too high a dose. Treatment with erythromycin is therefore recommended at a daily dosage not to exceed 125–250 mg × 2/day. Concomitant use of erythromycin and colchicine is not recommended due to the potential for potentiation of colchicine side effects.

Should erythromycin fail, one can try amoxicillin/clavulanic acid contained clavulanic acid which is prokinetic for the stomach.

Prokinetic treatment with metoclopramide or metopimazine PO may be proposed if there are no neurological and/or electrocardiographic contraindications.

Gastroparesis can lead to a state of severe undernutrition, requiring prolonged enteral (jejunal) feeding.

• Watermelon stomach

The medical treatment of watermelon stomach uses proton pump inhibitors. Endoscopic treatment (argon plasma coagulation, ND-YAG laser) may be necessary. If previous treatments have failed, antrectomy may be indicated.

Endoscopic monitoring must be done every 3 years.

• Intestinal disorders

b.** Motor disturbances causing malabsorption syndrome and/or pseudo-obstruction of the intestine**

In the case of acute occlusion, the first-line treatment consists of rehydration, analgesia (preferably avoiding opioids, which tend to exacerbate intestinal dysmotility), and relief of the small intestine by nasogastric aspiration. Nutritional management should not be delayed.

Intestinal prokinetic agents can be used.

Metoclopramide and domperidone often have little efficacy. The action of erythromycin on the motility of the small intestinal is less well known.

Intravenous neostigmine can be used in acute episodes, but cardiac and cholinomimetic adverse events limit its use in frail patients.

Analogs of somatostatin, mainly octreotide, may be proposed (**off-label use**). The initial dosage is 50 μg twice daily, and can be increased to a maximum of 100 μg twice daily depending on response to treatment. Beyond this dosage, octreotide may have an antisecretory effect, which may be harmful. A combination of low-dose erythromycin and subcutaneous octreotide is possible. Long-acting octreotide may be a better tolerated alternative to the daily subcutaneous erythromycin/octreotide combination for relapse prevention. In case of tachyphylaxis (need to increase the dose of the drug gradually to obtain an effect quantitatively as important as when it was introduced), a 3–4-week “wash out” should be done to restore its effectiveness.

Prucalopride could also be used at the same time, but this treatment is not currently reimbursed.

c. **Malabsorption syndrome by chronic bacterial colonization of the small intestine**

This colonization can be confirmed by performing a glucose breath test before starting the treatment.

The treatment is based on monthly sequential oral antibiotic therapy with alternating courses of different treatments, or even periods without treatment. The alternation of antibiotic molecules is proposed in order to avoid the emergence of a multiresistant intestinal bacterial flora. The durations are 10–14 days per month, and an alternation of three antibiotic molecules from different families is usually proposed. Commonly used antibiotics are amoxicillin (500 mg × 3/day), Noroxin (400 mg × 2/day) or ciprofloxacin (250 mg × 2/day) or other quinolones, doxycycline (100 mg/day) (and other tetracyclines), metronidazole (250 mg × 3/day), gentamicin (80 mg/day) or neomycin (500 mg × 4/day), and sulfamethoxazole 800 mg—trimethoprim 160 mg (1 tab 2× per day).

d. **Colonic disease**

The treatment of constipation is based on hygieno-dietary measures (balanced diet of fiber and mucilage, satisfactory hydration, regular physical activity), laxatives, and evacuating enemas. The advice of the nutrition team and/or a dietician is recommended for severe forms.

Prokinetic drugs can be combined to improve colon motility (and to a lesser extent symptoms): metoclopramide 20–30 mg daily, domperidone (the maximum daily dose is currently 30 mg daily in three doses) and prucalopride once daily (2 mg before the age of 65, and 1 mg over). Their use must be limited in time.

e. **Rectal prolapse and fecal incontinence**

The treatment of anal incontinence is primarily preventive. Prolonged and iterative pushing efforts should be avoided in patients with chronic constipation; biofeedback therapy can then be used.

The treatment of rectal prolapse is surgical.

In forms that are very disabling in everyday life, a stoma can be discussed.

f. **Undernutrition**

The causes of undernutrition are multifactorial. Oral food intake is often reduced due to persistent symptoms such as nausea, vomiting, reflux, and early satiety caused by intestinal dysmotility. Pullulation can cause poor digestion and poor absorption of specific nutrients. In addition, extraintestinal factors of malnutrition are often present: a low-residue diet with reduced mineral and vitamin intake, often due to mechanical factors and pain caused by the ingestion of residues (fruits and vegetables); low nutrient intake due to esophageal motor disorders. In addition, orofacial problems (microstomia, limited mouth opening, masticatory pain, dry syndrome, etc.) and contractures of the fingers can make it difficult to prepare and eat meals. The frequent anxiety and depression syndrome and the side effects of certain concomitant therapies (such as calcium channel blockers, prostaglandin derivatives, immune inhibitors, and opioids) may reduce appetite.

The management of undernutrition depends on its mechanism.

If a swelling is confirmed, it will be treated and prokinetic treatments put in place to improve intake and nutrient absorption. If the patient remains malnourished or symptomatic despite drug therapy, enteral nutrition, either by temporary nasal feeding or gastrostomy, should be discussed with an experienced team of gastroenterologists/nutrition specialists. In cases of severe gastroparesis, enteral feeding should be jejunal rather than gastric. If enteral feeding is not feasible or is ineffective, parenteral nutrition should be preferred. In all cases, schedule a consultation with a physician nutritionist or a dietitian specializing in SSc.

• **Orofacial diseases**

The management of oral manifestations is multidisciplinary, therapeutic, but above all preventive, particularly concerning the prevention of oral cavities and periodontal diseases. This is achieved by:

- Raising patient awareness of the importance of oral hygiene by teaching suitable brushing methods to prevent the appearance of cavities and periodontal lesions (gingivitis, periodontitis, gingival hyperplasia, etc.), taking into account the difficulties encountered by the patient. After a first oral check-up at diagnosis (combining clinical and radiological examination), biannual follow-up of the same type is recommended to detect any abnormality early.
- Oral care sessions should be at the beginning of the day and should be of short duration.
- Hyposialia should be managed by prescribing saliva substitutes and stimulators (pilocarpine hydrochloride if Sjögren’s syndrome is associated) to reduce the risk of periodontal diseases and cavities. Daily baking soda mouthwash should also be offered. Systematic fluoridation using fluorine-carrying devices or by applying fluoride varnishes should be discussed.
- In the event of mouth ulcers, application of topical antiseptics and anesthetics (such as chlorhexidine and lidocaine 2%) can be considered by avoiding the use of anesthetic products before meals to limit the risk of aspiration.
- Microstomia is one of the limits on the realization of care and may limit the making of impressions for prosthetic rehabilitation. Orofacial physiotherapy sessions should be offered to reduce the progressive limitation of the mouth opening. The technique of so-called fractured prints may be used in some cases. In addition, the base of the prostheses can be made of “soft” resin to limit the discomfort due to prostheses made of rigid materials (ulcers, pain, etc.).
- The use of dental implants depends on the patient. The practitioner must evaluate: on the one hand, the patient’s state of immunodepression, particularly in relation to therapeutics, but also the patient’s ability to maintain good oral hygiene so as not to compromise the viability of the implant. Posterior implants are used as often as possible, provided there is a sufficient mouth opening (Table 8).

**Table 8:** Orofacial diseases in SSc and appropriate oral management

Manifestations	Treatments
**Mouth and dental diseases**	**Prevention:** Pretherapeutic assessment Specific oral hygiene education Biannual screening: cavities and periodontal evaluation, check of mucous membranes and jaw bones Management of hyposialia **Specifics of care:** Short morning oral opening physiotherapy session Infectious risk to be taken into account in case of invasive procedure Indication for implant-dependent patients—prosthetic management-specific information

#### Cell therapies

• **Therapeutic intensification and autologous peripheral stem cell transplant**

The indications for bone marrow transplant in SSC are validated in Europe (EBMT and EULAR) and North America (CIBMTR) following the results of three randomized trials (ASSIST, ASTIS, and SCOT) that demonstrated with a grade 1 level of evidence the efficacy of the procedure in the short, medium, and long term with lower morbidity and associated mortality rate than observed with cyclophosphamide IV. Provided that patients are rigorously selected before receiving a transplant according to the EBMT international recommendations updated in 2017, the modalities of the procedure of therapeutic intensification followed by autologous hematopoietic stem cell transplant must be discussed on a case-by-case basis in the MCM to validate both the indication for and the absence of contraindications to the transplant in accordance with the good clinical practices (GCP) of the Société Francophone de Greffe de Moelle et de Thérapie Cellulaire (SFGM-TC) (Appendix 4).

• **Mesenchymal stem cell transplant**

This experimental cell therapy is based on the antifibrotic, trophic, angiogenic, and immunosuppressive properties of mesenchymal stem cells, which can be of hematopoietic or adipocytic origin. It is currently in trial phase 1 of 2. This procedure is not recommended by the working group (off-label use).

•** Injection of stromal vascular fraction**

The stromal vascular fraction corresponds to all the cells contained in the vascularization of adipose tissue. This includes endothelial progenitors, pericytes, mesenchymal multipotent progenitors, and leukocytes. It is an advanced therapy medicinal product (ATMP), which is currently being evaluated in a phase II clinical trial for the management of hand disability in patients with SSc. This procedure is not currently recommended by the working group.

**Extracorporeal photochemotherapy:** a prospective randomized study showed that extracorporeal photochemotherapy was more effective than D-penicillamine on skin lesions in SSc. However, these results are controversial. The working group does not recommend extracorporeal photochemotherapy in SSc.

### Functional re-education and rehabilitation

• **General and specific objectives**

Functional re-education and rehabilitation are part of the medical treatment of patients with SSc and should be prescribed as early as possible. Their general objective is to enable patients to maintain or increase their daily levels of activity and their participation in family, social, professional, and leisure activities.

The specific objectives of functional re-education are to prevent or reduce the impairments frequently encountered in the course of SSc, distinguishing between:

- Specific impairments: cutaneous, cardiorespiratory, musculoskeletal, and oral;
- Nonspecific impairments: deconditioning to effort, fatigue, anxiety, and depression.

Associated with functional rehabilitation, re-education also aims to prevent and reduce disabilities and to promote the maintenance or reintegration of patients with SSc in the family, social, professional, and leisure spheres.

Finally, the management of the functional re-education and rehabilitation program must enable patients to better understand their disease and to become actively involved in their treatment.

- **Means**

Functional re-education and rehabilitation involve multidisciplinary care and require the skills of different professionals: doctors, masseur–physiotherapists, occupational therapists, podiatrists, orthoprosthesists, dieticians, social workers, psychologists, and adapted physical activity teachers.

Functional re-education and rehabilitation programs are initially given in a supervised manner during sessions conducted under professional supervision, and then in an unsupervised manner during sessions conducted by the patients in their home.

The programs must be customized according to the results of the initial assessment and then according to the evolution during follow-up. To improve patient adherence to functional re-education and rehabilitation programs, they must be developed by taking into account the patient’s environment and preferences. Digitally supported programs, which are currently being tested in randomized studies, could eventually be made available to patients.

These means are:

**Re-education means**

- Massage and skin mobilization: palpate-roll on the most sclerotic skin areas to soften the skin;
- Joint mobilizations: passive and active mobilization, self-mobilization, postures, and self-postures to preserve joint mobility and prevent the onset of musculotendinous retractions;
- Muscle strengthening: muscles mobilizing stiff joints, to maintain the gain in amplitude obtained by passive mobilization techniques, and antigravity muscles to facilitate transfers;
- Functional work: transfers, walking, activities of daily living;
- Hand orthotics: resting orthoses which are intended to prevent deformations and are preferably worn at night, and dynamic (winding and extension) orthoses which are intended to correct deformations and to maintain or gain mobility and are preferably worn fractionally, two or three times a day for about 15 min, to postulate the fingers in flexion and extension. In case of skin ulcers on the dorsal side of the fingers or calcinosis on the pulpal side, orthoplastics (foam pads) can be used to improve the tolerance of the orthosis;
- Foot orthotics: their aim is to improve comfort while walking. The soles are made of flexible material and have pain-relief areas. Orthoplastics (postural and/or protective) aim to combat skin retraction.

**Rehabilitation means**

- Aerobic training;
- Adapted physical activity;
- Adapted technical aids: thick foam sleeves, systems to open bottles, jars, and cans more easily;
- Home furnishings: adaptation of handles, raising the bed and chairs, installation of electric shutter openers;
- Education: helps patients better manage their disease by developing their coping and self-care skills.

**Reintegration means**

- Social work: the social worker carries out the socioprofessional evaluation and accompaniment of patients to direct them towards the means and organizations that are best suited to achieve their life project.

## Follow-up

### Objectives

- Specify the activity and severity of the disease
- Screen for subclinical visceral damage
- Evaluate the effectiveness and tolerance of treatments
- Look for possible comorbidity
- Smoking cessation if active or passive smoking

### Professionals involved

The consultations required in the course of care depend on the initial assessment and on the progress:

- The general practitioner
- The reference and/or competence center
- Consultations with specialists

### Frequency and content of the consultations

The frequency of these consultations and examinations should be adapted to:

- The patient’s clinical status
- The severity and progression of the disease under treatment
- The treatments used (monitoring, tolerance, adverse effects)

**Clinical examination**

The follow-up clinical examination is the same as the initial assessment. The frequency of consultations is adapted to the clinical evolution. Generally speaking:

- In cases of diffuse cutaneous SSc diagnosed less than 3 years ago, quarterly clinical monitoring is indicated.
- In the case of limited cutaneous SSc in the absence of visceral involvement, biannual clinical monitoring is indicated.
- In the case of SSc with anti-RNA polymerase III Abs, and especially in patients 60 years old and above with a diffuse cutaneous form with duration of less than 3–5 years, the clinical examination should focus on looking for signs suggestive of gynaecological or solid cancer, at least during the first year following the diagnosis.
- In cases of diffuse cutaneous SSc diagnosed within the last 3 years, or if there is a risk factor for scleroderma renal crisis (initial edematous form, corticosteroid use, history of pericarditis, anti-RNA polymerase III Abs positivity), blood pressure monitoring by self-measurement methods is recommended at the onset of the disease to detect the occurrence of hypertension, which should suggest a possible scleroderma renal crisis and therefore lead the patient to consult a healthcare professional to consider confirmation of the hypertension and a laboratory test (see Sect. 5.5.8).

However, the frequency of consultations varies according to the initial severity and type of visceral damage and/or the occurrence of intercurrent events. A physical examination is needed with every modification of treatment.

During each clinical examination, particular attention should be paid to:

- Nutritional status
- Stature growth and pubertal development in children
- The functional aspect of skin and joint damage. Indeed, an evaluation of skin and tendon retractions and joint amplitudes must be carried out so that physiotherapists and re-educators can tailor their programs as best as they can.

**Paraclinical examinations**

The frequency of these exams, as well as the prescription of other complementary biological examinations, shall be adapted to:

- The patient’s clinical status
- The activity and severity of the disease
- The treatments prescribed (monitoring, tolerance, side effects)

Only those examinations that are essential for the screening and follow-up of complications and visceral damage that typically occur during SSc will be detailed.

Other examinations may be carried out according to the evolution of each patient. Additional tests specific to the monitoring of PAH when present are detailed in the PAH NDCP (HAS website, www.has-sante.fr/).

Systematic biological exams at each visit, adapted to the rhythm of clinical follow-up previously defined:

- CBC-platelets
- Reticulocytes, schizocytes, haptoglobin, LDH in case of suspicion of scleroderma renal crisis
- Blood electrolytes, creatinine, uric acid, CRP
- Albumin, hepatic assessment (AST, ALT, γGT, total bilirubin, and alkaline phosphatases)
- CPK
- Urine strip (possibly urine culture and protein-to-creatinine ratio on a sample)
- Antinuclear antibodies and antibodies specific to SSc should be repeated at 6–12 months in the absence of antinuclear antibodies in the initial workup or in the case of antinuclear antibodies without specificity as they may be secondarily positive. Apart from this particular situation, antinuclear antibodies are not a marker of the evolution of the disease. They should not be repeated systematically.

Depending on the objective deficiencies, previous results, clinical signs, current treatments, and the time between two exams (to be done at least once a year):

- Fasting blood glucose, calcemia, CPK
- Protein electrophoresis
- NT-proBNP (or BNP)
- TSH
- Ferritin level
- Folic acid and vitamin B12

A screening or follow-up check-up for visceral complications is generally carried out annually, possibly more frequently for recent diffuse skin forms.

- Respiratory function tests with DLCO

Annually or more often in cases of ILD and/or PAH that have already been diagnosed. Arterial blood gas is not always measured. It is done in case of dyspnea. In the case of blood gas, perform an Allen maneuver beforehand to ensure that there is no occlusion of the ulnar artery (frequent during SSc) which could make it dangerous to perform a radial arterial puncture (risk of radial occlusion leading to occlusion of both arterial trunks of the hand).

- High-resolution thoracic CT scan
- Systematic for unexplained acute or subacute exacerbation of ILD
- Otherwise, to be carried out in case of clinical changes, functional worsening, or changes on chest X-ray/scan
- Bronchoalveolar lavage
- No indication of systematic BAL during follow-up of ILD
- Should be performed during the evolution of the disease if there is any doubt about the evolutionary nature of the ILD or an infectious complication
- Annual cardiac ultrasound with myocardial function and measurement of VTI and PAPs to screen for PAH
- Right heart catheterization for diagnostic confirmation if pulmonary hypertension is suspected and then in case of proven PAH, depending on the clinic, during any discussion of a change in therapy and within 4–6 months of any change in therapy
- For some teams, a 6-min walking test, saturation, and Borg index are proposed as follow-up, even in absence of PAH
- EKG annually or more frequently if symptoms occur
- Holter monitor at the slightest suspicion of rhythm or conduction disorder
- Renal ultrasound, renal puncture, renal biopsy in case of suspected scleroderma renal crisis
- Gastrointestinal endoscopies:

In cases of dysphagia, worsening GERD, GI bleeding, or iron deficiency anemia

Systematic in patients with Barrett’s esophagus:

- If no dysplasia, monitor every 3–5 years
- Annually if high-grade dysplasia

Other gastrointestinal tests: only in symptomatic patients or depending on the context

In the case of SSc with anti-RNA polymerase III Abs, and especially in patients 60 years old and above with a diffuse cutaneous form of duration less than 3–5 years old, the working group proposes to carry out noninvasive screening tests for gynecological cancer (breast, ovarian, and uterine in particular), PSA assay in men, and a hemoccult^®^. The duration of this monitoring is not defined, although the increased risk of developing cancer is not demonstrated after 2–3 years after diagnosis. The appropriateness of more comprehensive or invasive examinations for neoplasia (cervical-thoraco-abdomino-pelvic CT and PET scan) has not been studied and is not recommended to date.

## Management of systemic sclerosis during pregnancy

### Sexuality and fertility of patients with SSc

The systemic nature of the disease can have consequences for a patient’s sexual life. Fatigue, depression, GERD, and change in physical appearance can also impact psychological well-being and interpersonal relationships. When these factors are taken into account, the data in the literature suggest that the fertility of women with and without SSc is similar. Cardiorespiratory or muscular damage can limit the ability to exercise. Cutaneous and articular changes and peripheral vascular damage are responsible for functional limitations (dyspareunia, digital ulcers, joint stiffness, etc.).

If immunosuppressive therapy is required for SSc during pregnancy, only azathioprine is permitted.

### Impact of SSc on pregnancy

- *Spontaneous miscarriages*: the overall rate of spontaneous miscarriages (spontaneous abortion before 22 weeks) in patients with SSc is between 12–15% of pregnancies and does not differ from that of the general population.
- *Premature births*: the rate of premature births (before 37 weeks) in patients with SSc varies between 11% and 40% of pregnancies. The risk factors for premature births are: recent diffuse cutaneous form (< 4 years), pulmonary disease, gastrointestinal disease, corticosteroid use, and intrauterine growth retardation. However, these premature births are most often induced.
- *Intrauterine growth retardation (IUGR)*: the proportion of children with IUGR (weight < 10th percentile at term) of mothers with SSc is between 5% and 6% versus 1–2% in the general population. Regular biometric ultrasound monitoring during pregnancy is therefore recommended.
- *Perinatal morbidity and mortality*: overall, there does not appear to be a difference in perinatal morbidity and mortality among women with SSc.

SSc does not appear to increase the risk of eclampsia, but any new occurrence of PAH should bring up a discussion about either eclampsia or scleroderma renal crisis. Regular BP monitoring should be performed during any scleroderma pregnancy as in a normal pregnancy, but at more regular intervals.

### Impact of pregnancy on SSc

Pregnancy does not appear to influence the course of SSc. Pregnancy increases gastroesophageal reflux. The systemic nature of the disease justifies a pre-conception consultation, followed by multidisciplinary treatment by trained teams.

- *Scleroderma renal crisis*: this is the most feared complication but remains rare. The factors favoring scleroderma renal crisis are mainly diffuse cutaneous forms that have been evolving for less than 3–5 years. It is difficult to diagnose a scleroderma renal crisis from preeclampsia in a patient with SSc (hypertension, hemolytic anemia, thrombocytopenia, proteinuria, acute renal failure). The lack of hepatic cytolysis and the rapid worsening of renal failure may lead to a scleroderma renal crisis. A kidney biopsy may help with the diagnosis, but will only be performed if it has an impact on maternal and fetal management. In the event of scleroderma renal crisis, treatment with an enzyme-converting enzyme inhibitor (ACEI) despite the fetal risk must be initiated as soon as the diagnosis is made.
  Chronic renal failure with glomerular filtration rate <30 mL/min/1.73 m^2^, proteinuria >1 g/24 h, and/or uncontrolled hypertension are contraindications to pregnancy.
- *Cardiopulmonary damage*: monitoring the respiratory function tests and the oxygen saturation is recommended. There is no consensus on limit values, but it seems reasonable to advise against pregnancy in cases of severe restrictive insufficiency (forced vital capacity < 50% of the reference value), and/or heart failure in NYHA class III or IV or with ejection fraction < 40%.
- *Pulmonary arterial hypertension*: according to ESC/ERS 2015 recommendations, PAH is a formal contraindication to pregnancy.

### Management during pregnancy

#### Pre-conception consultation

Programming a pregnancy in a patient with SSc should be considered during a pre-conception consultation. The primary aim of this consultation is to define the contraindications to pregnancy (respiratory, cardiac, and renal insufficiency, PAH, diffuse edematous form of recent onset).

It allows the adaptation of the DMARD (stopping an ACE inhibitor, replacing a teratogenic immunosuppressant by azathioprine, the only immunosuppressant authorized during pregnancy). The use of proton pump inhibitors, histamine H2-receptor antagonists, and calcium channel blockers for gastrointestinal and vascular disease is authorized (see NDCP systemic lupus 2017 and the Teratogen Reference Centre website) www.lecrat.fr). It ensures that the patient is immune to rubella, vaccinate her if necessary (if there are no contraindications), update other vaccinations, and start folic acid supplements.

A low dose of aspirin is allowed, but not routinely and depending on upper digestive tract involvement. Use of oral corticosteroids should be avoided as much as possible and should not exceed 10–15 mg/day.

A complete clinical and paraclinical check-up before the beginning of the pregnancy is desirable: skin assessment, baseline cardiopulmonary assessment, complete biological and immunological assessment (thyroid function, vitamin deficiencies, and if not previously done: anti-topoisomerase I Abs, anti-RNA polymerase III, anti-SSA/SSB, and antiphospholipids).

#### During pregnancy

In addition to monthly gynecological monitoring (fetal biometrics, uterine, and umbilical doppler), the aim is to look for signs of the disease’s progression. It therefore includes, periodically:

• Clinically, on a monthly basis:

- An assessment of dyspnea, signs of heart failure, skin disease
- Blood pressure, urine test strip (preeclampsia or scleroderma renal crisis)

• On a biological level:

- Creatinine, proteinuria
- Transaminases, gamma-GT
- Platelets, hemoglobin (or even LDH, haptoglobin)
- Blood sugar, toxoplasmosis serology if negative
- **On the ultrasound level:**

In addition to the three mandatory fetal ultrasounds (at 12, 22, and 32 weeks), the exam should be repeated more frequently, especially in patients with diffuse cutaneous and/or systemic damage, or if there is history of obstetrical problems. These ultrasounds will be supplemented by uterine and umbilical dopplers starting at 22 weeks.

• Therapeutically:

Certain maternal or fetal situations during pregnancy may warrant the administration of high doses of corticosteroids, either nonfluorinated or fluorinated. In the context of systemic sclerosis, taking into account the risk–benefit balance and the potential risk of a scleroderma renal crisis, these treatments should be discussed on a case-by-case basis.

These treatments are proposed in particular in situations of severe immunological or gestational thrombopenia and to prevent complications of prematurity by the administration of fluorinated corticoids (betamethasone 12 mg D1, D2, equivalent to a 160 mg total dose in prednisone), indicated between 24 and 34 weeks and in cases of risk of imminent delivery.

#### Childbirth and postpartum

An anesthesia consultation should be arranged early, especially if the mouth opening is reduced, in order to anticipate any difficulty in orotracheal intubation. Aspirin is not considered a contraindication to epidural analgesia, but may be discontinued at 35 weeks.

#### Breastfeeding

Breastfeeding is usually possible, provided that treatments are compatible, which is most often the case (CRAT website: www.lecrat.fr).

## Systemic sclerosis in children

SSc is extremely rare in children. The median age at first sign is 8.1 years (extremes 0.4 and 15.6 years), and a history of familial autoimmune disease is found in 10% of cases. Invoking this diagnosis requires specialized advice from the network of centers of reference and centers of competence for rare diseases. The diagnostic criteria for SSc are the same as the adult criteria (Table 1). Raynaud’s phenomenon is very frequent, often inaugural (70–95%), and precedes the other signs by an average of 19 months.

Manifestations of scleroderma (edema of the fingers, sclerodactyly, cutaneous sclerosis) are more likely to be found in the context of mixed connective tissue diseases associated at different levels with muscular damage, Raynaud’s phenomenon, and joint or cardiovascular manifestations. Renal crisis is exceptional, and PAH is reported in 3–14% of cases. Anticentromere Abs are exceptional in children.

The treatment is no different from that used for systemic forms in adults, although high-dose corticosteroid therapy is given more frequently in children, especially for skin diseases. It is well tolerated and does not appear to cause the complications (scleroderma renal crisis) encountered in adults.

The 5-year survival rate is about 85–90%. The causes of death are heart failure, PAH, kidney failure, respiratory failure, and infections. There is no long-term prospective study to assess long-term progress.

Because of its rarity, the main issue in pediatrics is differential diagnosis. Indeed, some genetic diseases can be associated with a scleroderma state (without skin ulcers) and lead to misdiagnosis. They include:

- Nodulosis arthropathy and osteolysis (NAO) syndrome, an autosomal recessive disease secondary to a mutation of the metalloproteinase 2 (MMP2) gene, combining generalized palm and plant nodules, early-onset arthropathy of the hands and feet, fusiform appearance of the fingers, and osteolysis of the carpus and tarsus.
- Laminopathies
- STAT3 gain-of-function mutations: the scleroderma aspect of the skin is associated with an immune deficiency and lymphoproliferation. There are no skin ulcers or inflammatory syndromes in these situations.
- Werner’s syndrome is an inherited syndrome caused by a mutation in the *WRN*s gene coding for one of the five RecQs of the human helicase family. It is characterized by early aging that appears between the ages of 20 and 30 with the main characteristics of bilateral cataract, short stature, and prematurely gray and fine hair. The retracted aspect of the fingers may look like SSc, but there is no Raynaud’s phenomenon and no skin infiltration.
- Other monogenic dysimmunitary diseases can promote the occurrence of true SSc, such as certain interferonopathies including spondyloenchondrodysplasia (*ACP5* mutation that codes for resistant tartrate acid phosphatase). Onset at very young age and association with other clinical and/or biological abnormalities must lead to a genetic cause.

## Figures and Tables

**Fig. 5:**
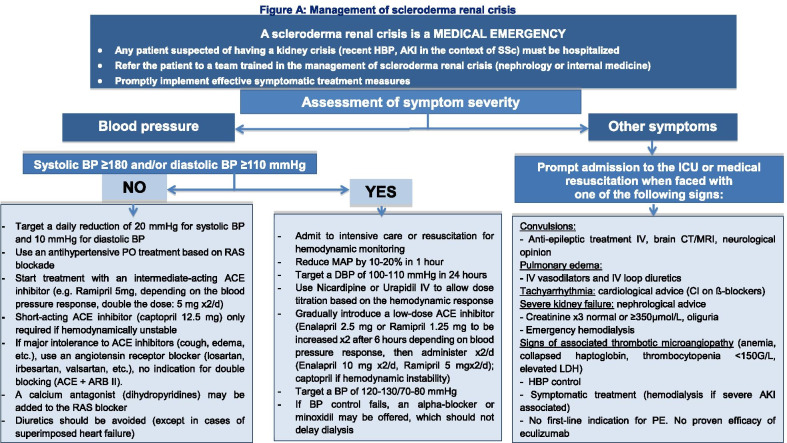
Management of scleroderma renal crisis

## Appendix 1: Modified Rodnan skin score (mRSS)

This score evaluates 17 points of the body by simple skin palpation and determines the extent of its thickening as follows:

0 = normal skin thickness

1 = minimal thickening

2 = moderate thickening

3 = severe thickening

It is a noninvasive score that is easy to perform but requires training and is validated as a prognostic marker by several studies (Clements et al. 1993). The total is out of 51.

**Table:**

	RIGHT				LEFT			
Fingers	□ 0	□ 1	□ 2	□ 3	□ 0	□ 1	□ 2	□ 3
Hands	□ 0	□ 1	□ 2	□ 3	□ 0	□ 1	□ 2	□ 3
Forearms	□ 0	□ 1	□ 2	□ 3	□ 0	□ 1	□ 2	□ 3
Arms	□ 0	□ 1	□ 2	□ 3	□ 0	□ 1	□ 2	□ 3

**Table:**

Thighs	□ 0	□ 1	□ 2	□ 3	□ 0	□ 1	□ 2	□ 3
Legs	□ 0	□ 1	□ 2	□ 3	□ 0	□ 1	□ 2	□ 3
Feet	□ 0	□ 1	□ 2	□ 3	□ 0	□ 1	□ 2	□ 3

**Table:**

Face	□ 0	□ 1	□ 2	□ 3
Anterior side of the thorax	□ 0	□ 1	□ 2	□ 3
Abdomen	□ 0	□ 1	□ 2	□ 3

To learn how to perform the modified Rodnan score in practice, click the link: https://www.youtube.com/watch?v=IUtCq3JPfAc&feature=youtu.be

## Appendix 2: Rating scales

Cochin Hand Functional Scale (Rannou et al. 2007)

Scleroderma HAQ (SSc-SHAQ) (Georges et al. 2005)

Check each of the devices you use regularly:

Check the items for which you usually need someone’s help:

How intense has the pain from your disease been this past week?

(make a mark on the line to indicate, on a scale of 0 to 100, the intensity of the pain)

- **SF 36** (Georges et al. 2006)

*9. Here is a list of activities that you may have to do in your everyday life. For each of them, indicate whether you are limited on account of your current state of health*.

(*circle the answer of your choice, one per line*)

**Table:**

	List of activities	Yes, very limited	Yes, a little limited	No, not at all limited
a	Major physical efforts such as running, lifting a heavy object, playing sports	1	2	3
b	Moderate physical efforts such as moving a table, vacuuming, bowling	1	2	3
c	Going shopping	1	2	3
d	Climbing several flights of stairs	1	2	3
e	Climbing one flight of stairs	1	2	3
f	Bending over, kneeling, squatting	1	2	3
g	Walking more than a kilometer	1	2	3
h	Walking several hundred meters	1	2	3
i	Walking a hundred meters	1	2	3
j	Taking a bath, a shower or getting dressed	1	2	3

*10. The following questions are about how you have felt over the past 4 weeks. For each question, please indicate the answer that seems most appropriate to you. Over the past 4 weeks, have you had moments when*:

(*circle the answer of your choice, one per line*)

**Table:**

	Constantly	Very often	Often	Sometimes	Rarely	Never
a. Have you felt dynamic?	1	2	3	4	5	6
b. Have you felt very nervous?	1	2	3	4	5	6
c. Have you felt so discouraged that nothing cheers you up?	1	2	3	4	5	6
d. Have you felt calm and relaxed?	1	2	3	4	5	6
e. Have you felt overflowing with energy?	1	2	3	4	5	6
f. Have you felt sad and dejected?	1	2	3	4	5	6
g. Have you felt exhausted?	1	2	3	4	5	6
h. Have you felt happy?	1	2	3	4	5	6
i. Have you felt tired?	1	2	3	4	5	6

*11. Indicate for each of the following phrases to what extent they are true or false for you*:

(*circle the answer of your choice, one per line*)

**Table:**

		Somewhat true	I don’t know	Somewhat completely	
	Completely true			False	False
a. I get sick more easily than others	1	2	3	4	5
b. I am doing as well as anybody	1	2	3	4	5
c. I expect my health to deteriorate	1	2	3	4	5
d. I am in excellent health	1	2	3	4	5

Please make sure you have provided an answer for every question.

Thank you for your collaboration.

*Copyright © New England Medical Center Hospitals, Inc., 1993 All rights reserved. (IQOLA SF-36 French (France) Version 1 3)*.

## Appendix 3: Specifics of the local management of systemic sclerosis digital ulcers (DU) and choice of dressings

### Specifics of the local management of digital ulcers (DU)

(According to Lok et al. 2011).

1. **Local management**
  Little research has been done on the specific local management of DUs in SSc; it is mainly based on the experience of practitioners. Hygiene care of DUs is no different from other chronic wounds: wash with soap and water or saline to remove surface germs and dressing residues. The daily use of antiseptics on these chronic wounds should be avoided, except in the case of infection.
2. **Choice of dressing (tables below: use of different classes of dressings and choice of dressing)**
  As with any chronic wound, the dressing must be adapted to the stage of the wound. Nevertheless, the location of the DUs requires some adaptations. Practical, small size dressings that fit easily on the fingers should be chosen to improve patient comfort by making it easier for the patient to move around in their everyday life. It is therefore necessary to give preference to conformable dressings (film or extra thin, finger, thin multilayer dressings) and nonadherent dressings. Hydrocellulars, hydrofibers, and hydrocolloids can be used. Hydrogels, some of which are conformable, and hydrofibers can be used for detergency. Thin dressings with tulle or interfaces are practical when the DU has been cleaned, as they avoid the accumulation of multiple layers of primary and secondary dressings. Creams, such as hyaluronic acid, are easy to use and painless to remove, but must be covered with a secondary dressing. As a secondary dressing, the use of plastic finger pads can be useful and practical in active patients.

In the case of necrotic DUs, antisepsis is required, followed by a dry dressing to allow the necrosis to dry. Surgical advice is required to consider limited amputation of the necrotic area, but self-amputations are common. On sprouting DUs, skin grafts in pellets or small fillets can be offered for healing and analgesic purposes.

3. Cutaneous sclerosis and cutaneous xerosis slow down the healing process and can lead to new wounds, especially in flexural areas. It is therefore necessary to moisturize by applying cold cream or neutral emollient several times a day and to quickly treat the cracks with repair and healing creams to avoid the evolution towards a DU *(see table creams with healing properties).*

Main dressings useful for DUs (nonexhaustive list from the *Practical Handbook of Prescription Drugs* (Dorosz. Ed Maloine 2017)

**Table:**

Types of dressing and specialty name	Notes	Conditions of use
**Hydrocolloids** Comfeel, Algoplaque, Duoderm, Tegaderm Hydrocolloid, Askina, etc.	Thick adhesive plates Thin, translucent adhesive plates Paste tubes	1 application every 2–7 days without a secondary dressing 1 application under a hydrocolloid plate for paste version
**Hydrocellulars** Allevyn, Biatain, Aquacel Foam, UrgoTul, Combiderm, Mepilex, etc.	Peripherally adhesive plates or peripherally reinforced or nonreinforced microadhesive plates Nonadhesive plates	1 application every 2–7 days, with secondary dressing if nonadhesive or non-reinforced form on the periphery
**Hydrocellular dressing with protease inhibitors** Urgostart	Nonadhesive or microadhesive plates Interface	1 application every 2–7 days (secondary dressing required)
**Alginates** Algosteril, Urgosorb, Melgisorb, Biatain	Sterile compresses, usually: 5 × 5, 10 × 10, 10 × 20, 15 × 15 cm	1 application every 1–2 days (secondary dressing required)
**Hydrogels** Duoderm, Hydrosorb, IntraSite Normlgel, Purilon, Nugel, Tegaderm Hydrogel, etc.	10 × 10 plaques Tubes, dosing applicator, prefilled syringe or 15 g bag	1 application every 48 h, secondary dressing required, low absorbent and waterproof (polyurethane film, thin hydrocolloid)
**Carboxymethylcellulose or polyacrylate fiber dressings** Aquacel; Urgoclean	Compresses	1 application every 1–2 days (secondary dressing required)
**Interface dressings** Adaptic, Urgotul, Physiotulle, Hydrotul, Mepitel, Curity, Mepitel One	Tulle sheets coated with or soaked in various non-adhesive products (carboxymethylcellulose, silicone, etc.)	1 application every 1–7 days (secondary dressing required)
**Silver dressings** UrgoTul Ag, UrgoCell Ag (nonadhesive or Border),	Interface (± compress; ± peripheral adhesive) Hydrocellular plate	1 application every 2–7 days (secondary dressing for nonadhesive types)
**Hyaluronic acid dressings** Ialuset	Cream tube 100 g, pressurized bottle 100 g, soaked tulle compresses 10 × 10 cm Adhesive hydrocolloid plate 10 × 10 cm	1 application/day (secondary dressing required unless plate type)
**Ointment dressings** Rassolind, Jelonet, Vaselitulle, Tulle gras, Cuticell, etc.	Plates, usually 10 × 10 or 20 × 20 cm	1 application every 1–2 days (secondary dressing required)
**Semipermeable adhesive films** Hydrofilm, Opsite, Lumiderm, Tegaderm, etc.	Transparent adhesive plates with or without integrated compresses	1 application every 1–7 days

Creams with healing properties, nonexhaustive list (hands and cracks)

**Table:**

Trade name	Composition
Cicaplast (La Roche Posay)	Copper zinc manganese complex—Madecassoside
Cicalfate (Avène)	Copper zinc complex—Sucralfate
Epithelial AH cream (Aderma)	Hyaluronic acid—Rhealba oats extract—Vitamin A—Vitamin E
Cicabio (Bioderma)	Copper zinc complex—Resveratro l—Hyaluronic acid
Bépanthen ointment (Bayer)	Dexpanthenol 5% (possible allergies)
Bariéderm cream (Uriage)	Poly-2p

## Appendix 4: Indications and contraindications for autologous hematopoietic stem cell transplant in SSc

(According to Farge et al. 2017).

Indications for AHSC transplantation during SSc

Treatment with autologous hematopoietic stem cell transplantation may be discussed in patients with severe and progressive, life-threatening or organ-related SSc (each case should be discussed in MCM).

Contraindications common to AHSC transplantation in autoimmune diseases:

- Age > 65;
- Active or weaning smoking for less than 3 months
- Pregnancy: ongoing pregnancy or refusal to use effective contraception
- Psychiatric: psychiatric illnesses including alcohol or drug abuse
- Consent: inability to give informed consent for the choice of procedure
- Liver function:
  - Elevation of transaminases or bilirubin above twice the normal
  - Liver failure or confirmed cirrhosis
- Cancer: myelodysplastic syndrome or severe hematological disease contraindicating autologous hematopoietic stem cell transplantation
- Infections: acute or chronic infections related to HIV, HTLV-1, HTLV-2, hepatitis B or C
- Heart:
  - Left ventricular ejection fraction < 45%
  - PAPm > 25 mmHg or PAPs > 40 mmHg without any filling
  - PAPm > 30 mmHg or PAPs > 45 mmHg after filling test with injection of 1000 cc of isotonic saline over 10 min
  - Diastolic paradoxical movement of the septum (D-sign)
  - Paradoxical septum
  - Constrictive pericarditis
  - Cardiac tamponade
  - Marked atherosclerosis
  - Heart rhythm disorders that cannot be controlled pharmacologically or by cardioversion or ablation
- Lungs:
  - FVC < 65% of the reference value
  - DLCO < 40% of the reference value

Note: if echocardiogram, cardiac MRI, or cardiac catheterization with or without a fluid load test did not show contraindications before the AHSC transplantation, patients with a DLCO < 65% of the reference value or with a FVC < 40% of the reference value may be considered as candidates for the transplantation after being discussed in the MCM.

- Patients should be strongly encouraged to stop smoking;
- Renal:
  - Scleroderma renal crisis in the previous 6 months
  - Glomerular filtration rate < 40 ml/min/1.73 m^2^

Note: patients with history of renal crisis or renal failure may be considered candidates for transplantation if their blood pressure is under control and dialysis is performed the morning following each cyclophosphamide infusion

## Appendix 5: Absolute or relative contraindications to lung(/cardiac) transplants in systemic sclerosis

According to Launay et al. (2014).

**Table:**

Absolute contraindications:
Visceral failure outside therapeutic resources (especially renal failure with glomerular filtration rate < 50 ml/min/1.73 m^2^)
Extrapulmonary infection with no therapeutic resources to hope for a cure (hepatitis B virus infection, hepatitis C virus infection, HIV)
Active or recent cancer (less than 2 years) with the exception of basal cell or squamous cell skin cancers or cancer in situ, which would be completely resected. A period of 5 years is recommended before discussion of a possible transplant in cases of history of high-grade colon cancer, breast cancer, kidney cancer, or in cases of a melanoma of stage III or beyond
Active smoking or other addiction in the last 6 months
Uncontrolled psychiatric or psychological disorders with inability to cooperate or undergo regular medical treatment
Thoracic or spinal deformity or degenerative neuromuscular pathology likely to significantly interfere with mechanical ventilation
Documented nonadherence to therapy
Body mass index < 15 kg/m^2^
**Relative contraindications:**
Age > 65 years for mono- or bipulmonary transplants and age > 55 years for cardiopulmonary transplants
Severe or symptomatic osteoporosis
History of thoracic surgery
Undernourishment (15 kg/m^2^ > BMI < 17 kg/m^2^) or obesity (BMI > 30 kg/m^2^)
Invasive mechanical ventilation
Colonization with resistant bacterium, fungal, or mycobacterial agent
Severe loss of autonomy with low rehabilitation potential
Uncontrolled comorbidity (hypertension, diabetes)
**Specific contraindications related to systemic sclerosis:** *(Some may be temporary, if regression or improvement under treatment)*
Uncontrolled active inflammatory myopathy
Progressive myopathy
Diaphragmatic myopathy
Digital ulcers: More than one severe episode per year despite optimal treatment Active digital ulcer: temporary contraindication
Gastrointestinal: Esophageal stenosis Active peptic ulceration despite optimal therapy including PPIs and prokinetic drugs Barrett’s ulcer with high-grade dysplasia Gastroparesis (abnormal gastric emptying with less than 25% clearance at 90 min of ingestion) despite medical treatment Chronic gastrointestinal bleeding with or without anemia Specific small intestinal malabsorption or pseudo-occlusion type disorder Colorectal injury with pseudo-obstruction and/or diverticulitis and/or perforations
Heart disease: Conductive and/or rhythm disorders (symptomatic bradycardia, ventricular or atrial tachycardia): pretransplant management should be discussed (pacemaker if necessary), but it should not be a contraindication if cardiopulmonary transplantation is discussed.
Renal disease: Renal function must have been stable for at least 3 months except in situations of renal failure due to right ventricular dysfunction A period of at least 3 years must be respected in case of history of scleroderma renal crisis before discussing any lung or cardiopulmonary transplant Situations at risk for scleroderma renal crisis: Diffuse SSc evolving for less than 3–5 years since the first symptom outside Raynaud’s Rapidly progressive severe skin damage: increase in Rodnan’s score of at least 25% over the last 6–12 months Corticosteroid therapy > 15 mg of prednisone equivalent per day

*HIV* human immunodeficiency virus, *BMI* body mass index, *HT* hypertension

## Appendix 6: Recommendations for anesthetic and resuscitative management of the patient with systemic sclerosis

There are currently no published recommendations on general or regional anesthesia for patients with SSc. However, like any other patient, they may be exposed to situations of scheduled or emergency surgery. General anesthesia can sometimes be delicate due to the existence of difficulties with intubation linked to microstomia, the frequency of gastroesophageal reflux, which can sometimes be severe, and the existence of pulmonary or cardiac damage in some cases. Local, locoregional anesthesia, and even hypnosis are alternatives that should be discussed whenever possible. The technical problems encountered in perioperative situations and during acute medical complications in intensive care are often common and are therefore dealt with in the same section.

Risk assessment: cardiopulmonary and renal assessment

A preoperative check-up, particularly a cardiopulmonary check-up, is essential before any anesthesia of a patient suffering from systemic sclerosis.

• Pulmonary fibrosis

The search for pulmonary fibrosis must be systematically carried out by a thin-slice thoracic CT scan associated with respiratory functional explorations with measurement of the slow diffusion of carbon monoxide (DLCO). Anesthesia can be tricky if the forced vital capacity is < 50% of the predicted value due to difficulties in weaning the patient from mechanical ventilation if they were intubated (general anesthesia). Oximetry in ambient air and the realization of a blood gas allow to look for hypoxia if there is interstitial pulmonary damage.

• Pulmonary hypertension (PH)

Cardiac involvement is not uncommon during SSc, with primarily diastolic dysfunction of the left ventricle, linked to the existence of myocardial fibrosis and/or a decrease in coronary reserve in certain microcirculatory territories. In all cases, signs suggestive of PH should be looked for by echocardiographic screening and, if in doubt, or if there is an elevation in systolic pulmonary arterial pressure, request a right heart catheterization. In the case of PH, anesthesia is indeed riskier or even contraindicated. In the case of PAH (group 1 pre-capillary by involvement of the small pulmonary arteries), any surgical procedure must be discussed according to the hemodynamic situation (right heart catheterization) and discussed with the reference center or a competence center. These patients are usually nonresponders to inhaled NO. In this situation, local or locoregional anesthesia will be preferred, subject to constant and monitored maintenance of right heart preload. If, on the contrary, there is post-capillary PH on diastolic dysfunction, the prevention of pre-anesthetic decompensation accidents requires a preoperative reduction in pressure.

A routine baseline electrocardiogram is necessary to detect conduction disorders, rhythm disturbances, in addition to abnormalities related to pericarditis, left ventricular failure, and/or right heart failure.

• Renal disease and scleroderma renal crisis

To prevent the risk of acute renal injury, hydration should be optimized and nephrotoxic therapy should be avoided whenever possible. Special caution is advised for iodinated contrast media. Hemoglobin levels should be maintained high enough to promote good renal perfusion without excessively increasing blood viscosity to promote microcirculatory flow.

In the pre-anesthesia work-up, particular attention should be paid to ensuring that the blood pressure is normal, as patients with recent scleroderma that has been evolving for less than 3–5 years, especially diffuse cutaneous forms, are at higher risk of scleroderma renal crisis. A formula count, renal function with urea and creatinine, plasma ionogram, liver workup, coagulation workup, blood type, and proteinuria should also be performed.

Intubation risk assessment and airway preparation

Scleroderma skin damage frequently affects the face and can lead to nasal refinement and thus a reduction in the size of the nasal fossae, which can hinder the progress of an intubation tube inserted into a nostril. Skin damage will also reduce the mouth opening, which may be further reduced if there is fibrosis of the temporomandibular joint. It is therefore necessary to anticipate these intubation difficulties and to ensure that mask ventilation will not be a problem beforehand. Thinner intubation tubes should also be used if necessary. We recommend, when possible, in anticipation of a scheduled surgery, such as for dental care, physiotherapy to open the mouth in the previous month, which can result in a gain of 5–10 mm of mouth opening.

If, in a preoperative context, passage through the mouth appears difficult, any passage of an intubation tube through the nose must be done with great care because scleroderma patients may have telangiectasias of the mucous membranes, particularly of the upper aerodigestive tract, with a risk of bleeding during the progression of the tube. Decubitus intubation may also be hampered by frequent gastroesophageal reflux due to the cardiac gap. This implies the pre-anesthetic control of gastric emptying by ultrasound, even in case of prolonged preoperative fasting. Hyperextension of the head can also be difficult due to cutaneous sclerosis of the neck and can therefore limit visibility by obstructing the passage of the laryngoscope.

These elements of the pre-anesthetic examination should lead to the anticipation of using a fiberscope if a difficult intubation is expected. If intubation is not possible, a situation that is still very rare, a tracheotomy under local anesthesia should be considered.

Digestive and nutritional assessment

- **Gastroparesis**

Approximately half of scleroderma patients have gastroparesis, which manifests itself as early satiety, sometimes nausea, vomiting after meals with undigested food, and anorexia. The diagnosis can be made during gastric fibroscopy that finds food in the stomach while the patient is fasting. Chronic gastroparesis and pseudo bowel obstruction may contribute to a difficult and prolonged postoperative period. At the slightest doubt, a study of gastric emptying by gastric scintigraphy should be done in the preoperative check-up with late images at 3 or 4 h. Systematic intraoperative prevention of postoperative nausea and vomiting with Domperidone and Droleptan is recommended (be careful with dexamethasone because corticosteroids can promote the onset of a renal crisis, especially if it is a recent diffuse cutaneous SSc).

- **Nutritional disorders**

In the case of severe gastrointestinal problems (severe eso-gastric problems, lower gastrointestinal problems), there may be signs of undernutrition, vitamin deficiencies, hydroelectrolytic disorders. They need to be screened and treated.

Preoperative precautions

- **Transfusion and anticoagulation**

There is no specific recommendation for transfusions; they can be done in scleroderma patients if necessary depending on the type of surgery. In particular, a program of autotransfusion or erythropoietin treatment is possible as in any other patient for scheduled surgery at risk of bleeding (total hip replacement). Anticoagulants, if indicated, may increase the risk of gastrointestinal bleeding if peptic esophageal ulcers or gastroduodenal or colonic telangiectasias are present. An oesogastric fibroscopy is desirable before any surgery that would require effective anticoagulation. The presence of antiphospholipid Abs may suggest anticoagulation during the intraoperative period.

- **Interactions between anesthesia and medications taken by the patient**

Some patients take low-dose steroids and/or are on immunosuppressants. Therefore, they have an increased risk of postoperative infection. It is recommended to prescribe a systematic immediate preoperative antibiotic prophylaxis, the spectrum of which depends on the surgical procedure.

For patients receiving long-term corticosteroid therapy, adaptation to stress is limited by the slowing down of the corticotropic axis. A possible adrenal insufficiency must be supplemented by intravenous administration of 100 mg hydrocortisone hemisuccinate immediately before surgery and anesthesia.

WARNING: ACE inhibitors are the first-line antihypertensive treatment for patients with SSc with hypertension. They can induce refractory hypotension at the time of induction of anesthesia. Under anesthesia, the contribution of the renin–angiotensin system to blood pressure maintenance is important and may be altered by certain anesthetic agents. The action of angiotensin II being blocked by ACE inhibitors or AT-1 antagonists can therefore lead to the occurrence of hypotension, which can sometimes be severe and react poorly to catecholamines, particularly in cases of hypovolemia or under local anesthesia. This treatment must therefore be interrupted before the procedure. When such interruption is not possible, the occurrence of hypotension, either at the time of anesthetic induction or during hypovolemia, is more frequent. A simple treatment with an adrenergic agonist such as ephedrine is usually effective but can worsen digital ischemia.

Precautions in the operating room and in the ICU

Warming

The patient must be warmed up in permanence with a heating blanket in the operating room and during transport, especially at the extremities due to the high risk of digital ischemia (hands, feet). If possible, the temperature in the operating room, usually set at 18 °C, should be increased.

- **Installation in the operating room**

During the surgical procedure, the Trendelenburg position (patient lying on his/her back with lower limbs higher than the head) may aggravate the gastroesophageal reflux and should therefore be avoided (or even proscribed) if the patient is not intubated, due to the risk of aspiration. In patients with diffuse cutaneous SSc, cutaneous fibrosis associated with periarticular fibrosis may significantly impair joint mobility, particularly of the wrists, elbows, shoulders, hips, and knees. In addition, in patients with a diffuse form of scleroderma and possibly amyotrophy or subcutaneous calcifications, the pressure areas on the operating table must be protected because of the risk of pressure sores during long operations. In particular, direct contact of the patient’s skin with the metal parts of the operating table must be avoided and care must be taken to ensure that the electric scalpel plate is not too cold.

- **Recovery**

The patient should be particularly monitored in the recovery room because of the significant risk of vomiting associated with gastroesophageal reflux. It is not uncommon for scleroderma patients to have dry eye and dry mouth syndrome. It is also important to make sure that the oral cavity and the tongue are well hydrated, as they can quickly dry out when breathing through the mouth.

- **Anesthetic and procedural precautions: choosing the type of anesthesia**

When used preoperatively, hypnosis can reduce anxiety as well as postoperative nausea, vomiting, and pain.

Whenever possible, locoregional anesthesia is preferred, subject to strict control of the preload by appropriate noninvasive monitoring. Locoregional anesthesia is generally not a problem, as the spine is spared during SSc. For childbirth, obstetric analgesia is recommended. In the case of respiratory impairment, opioid drugs should be used sparingly. Locoregional anesthesia allows for prolonged postoperative analgesia.

Whenever general anesthesia is required, endotracheal intubation is recommended to avoid the risk of reflux and aspiration. Assisted ventilation can be tricky, and the ventilatory mode should be adapted to the decrease of pulmonary compliance in cases of pulmonary fibrosis.

- **General precautions**

The use of vasopressor amines may aggravate Raynaud’s phenomenon and the risk of digital necrosis. Their use must be limited as much as possible. In cases of deep digital ischemia, treatment with Iloprost IV should be offered as soon as possible depending on the hemodynamic status of the patient.

NSAIDs should be avoided because of the risk of gastrointestinal complications, the risk of peptic ulcer of the esophagus, the risk of gastrointestinal hemorrhage due to the presence of telangiectasias in some cases, and the risk of renal damage in certain patients, particularly those with a diffuse cutaneous form that has been evolving for less than 3–5 years.

The surgical procedure must be accompanied by gastrointestinal protection through the use of intravenous proton pump inhibitors (PPIs). In patients with gastroparesis, an intravenous injection of erythromycin at the time of induction of anesthesia allows the stomach to be emptied and limits the risk of inhalation.

Cardiorespiratory monitoring

Skin thickening and/or joint contractures can make it very difficult to establish a venous line.

Oxygen saturation should not be measured on the finger or ear as Raynaud’s phenomenon is frequent in the operating room and in acute situations, but on the forehead using a suitable sensor.

For lengthy procedures, it is recommended to avoid invasive blood pressure measurement, especially radial pressure measurement. The risk of this procedure is radial thrombosis and digital necrosis, or even acute ischemia of the hand if there is ulnar thrombosis (10–15% of patients).

General anesthesia is very delicate if there is pulmonary arterial hypertension due to the consequences of hemodynamic variations. Surgery in these situations must be discussed at length, and if it is to be performed in emergency situations, the advice of the reference center or a pulmonary arterial hypertension competence center is essential. In this situation, local or locoregional anesthesia will be preferred. For orthopedic procedures, cemented prostheses should be avoided because of the embolic risk at the time of nailing, which can increase pulmonary arterial hypertension.

Risks of complications

Patients with SSc are at greater risk for intubation failure and difficulty, GERD, and aspiration.

Patients with pulmonary fibrosis are at risk of hypoxia, desaturation, and reduced lung compliance, especially if there is thoracic skin involvement. There is therefore an increased risk of sudden desaturation in these situations, particularly when maneuvering in the upper airways.

Scleroderma patients may be hypersensitive to opiates and therefore at a higher risk of postoperative respiratory failure when forced vital capacity is < 1 L.

In case of cardiac damage with diastolic and/or systolic dysfunction, there is a risk of hypotension, which is sometimes severe, at the time of induction of anesthesia due to the induced vasodilatation. There may also be a risk of pulmonary edema with rapid filling if there is an unknown systolic and/or diastolic dysfunction.

Stress, pain, dehydration, hypothermia, and vasoconstrictive therapies increase the risk of vasospastic crisis and thus of distal ischemia and digital necrosis.

In the case of colic or small bowel disease, gastroparesis can be induced by the procedure, thus creating a risk of obstipation. In some cases, microbial pullulation can lead to abdominal bloating and diarrhea. Some patients may develop a pseudo bowel obstruction. Others may develop true occlusions if there is obstipation. In these situations, if there is the slightest doubt, an abdominal CT scan should be performed to look for obstipation or chronic intestinal pseudo-obstruction.

The concomitant use of low doses of corticosteroids and/or immunosuppressants increases the risk of postoperative infection.

Postoperative care

Postoperative care depends on the type of surgery and the severity of the disease. Postoperative ventilation is sometimes necessary because of the risk of postoperative respiratory failure in some patients. Temporary supervision in the ICU may be required. The same is true in patients with a specific heart disease or pulmonary arterial hypertension.

Continuous EKG monitoring is recommended in the postoperative period with volume and tension monitoring.

Postoperative analgesia is locoregional at best. NSAIDs should be avoided, and opioids should be used with caution.

Kidney function and blood pressure should be carefully monitored throughout the surgical procedure and in the days following it. There is indeed a risk of scleroderma renal crisis, especially in patients with a diffuse cutaneous form that has been evolving for less than 3–5 years. Early warning signs are the occurrence of high blood pressure (≥ 140/90 mmHg confirmed at least twice) and/or a rapidly progressive renal failure that may become oligoanuric. Biologically, microangiopathic hemolytic anemia with schizocytes should be investigated for the occurrence of microangiopathic hemolytic anemia with schizocytes and for decreased haptoglobinemia with elevated LDH.

Obstetrical particularities

In the vast majority of cases, women with scleroderma can give birth through the vaginal route. Epidural analgesia is possible and recommended in women with SSc. An episiotomy may be necessary for first-time deliveries, as in women without SSc. In certain situations of very pronounced sclerosis that may hinder the passage of the newborn through the birth canal, caesarean section may be indicated under local anesthesia.

The occurrence of a scleroderma renal crisis during pregnancy may appear as preeclampsia, but in the case of a renal crisis, the plasma renin level is very high, and renal failure is progressive and rapidly develops into microangiopathic hemolytic anemia. Emergency treatment is the use of ACE inhibitors, regardless of the creatinine level and of the stage of the pregnancy, due to the therapeutic emergency.

### Conclusion

Anesthesia and the perioperative period are situations with a high risk of morbidity and mortality in patients with SSc. A well-conducted preoperative check-up involving close collaboration with reference and competent centers enables optimal precautions to be taken during this period. The medical and technical specificities encountered in the perioperative situation can for the most part be extended to intensive care management for acute medical situations.

## Appendix 7: Emergency guidelines

This appendix has been extracted and modified from ORPHANET’s 2017 emergency guidelines on SSc. www.orpha.net/data/patho/Emg/Int/fr/Sclerodermie_FR_fr_EMG_ORPHA801.pdf

xii **Emergency guidelines for paramedics**

Mechanisms

Associated autoimmune disease: Raynaud’s phenomenon, cutaneous sclerosis and severe gastroesophageal reflux, a variable visceral involvement.

Special risks in emergencies

Malignant arterial hypertension, cardiac decompensation (on PAH or scleroderma cardiomyopathy), respiratory distress (on fibrosis or severe PAH), upper gastrointestinal hemorrhages

Frequently prescribed long-term treatments

- Raynaud’s phenomenon: calcium channel blockers
- Ulcer or digital necrosis: iloprost (antiplatelet agents)
- Heart disease or PAH: conversion enzyme inhibitor, diuretics, digitalis, low-salt diet
- Pseudo-occlusive syndrome: octreotide, erythromycin
- Pulmonary fibrosis, muscle damage: immunosuppressants
- Pulmonary fibrosis: respiratory physiotherapy
- PAH: sildenafil, tadalafil, bosentan, ambrisentan, intravenous epoprostenol, subcutaneous treprostinil, iloprost aerosol
- Painkillers

Traps:

- Decompensation of PAH during physical exertion, altitude, excess salt, or taking antiinflammatory drugs
- Risk of focal myocardial ischemia, rhythm disturbance or healthy coronary conduction on myocardial fibrosis

Specifics of pre-hospital medical care

- Difficult venous approach (skin is sometimes brittle, fibrotic veins)
- Patient is usually allergic
- Electrocardiogram indicated in initial assessment
- Urine test strip: existence and severity of a renal crisis
- On-board biology: kalemia, hemoglobin, troponin, gasometry to assess the impact of the SSc and referral to an intensive care setting
- Contraindication of nitrates or similar derivatives if treated with phosphodiesterase V inhibitors (sildenafil, tadalafil, etc.)
- Increased risk of bleeding for patients on iloprost
- Anti-aggregants need be adjusted according to etiology and ischemic risk
- Precautions for GA and the choice of the mode and parameters of invasive or noninvasive ventilation, depending on the systemic damage, in particular pulmonary or cardiac.
- Vasopressive amines capable of increasing peripheral vasoconstriction, especially digital vasoconstriction
- Risk of difficult intubation: limitation of the mouth opening, severe GERD

Guidelines for hospital emergencies

A. **Emergency recommendations**
B. **Emergency situation 1: Malignant hypertension on scleroderma renal crisis**

Scleroderma renal crisis is a complication that occurs more particularly in patients with recent (<3–5 years of evolution) and rapidly progressive diffuse cutaneous scleroderma.

It is associated with de novo hypertension and increased creatinine levels.

The presence of anti-RNA polymerase III ABs constitutes an over-risk of kidney failure.

It is a malignant HTA with retinal hemorrhaging and exudates.

Diagnosis is therefore generally easy (Table

**Table 9:** Criteria for the classification of the hypertensive form of scleroderma renal crisis

a. HBP ≥ 140/90 mmHg (or increase of systolic BP ≥ 30 mmHg or diastolic BP ≥ 20 mmHg) obtained on two different measurements with a minimum separation of 5 min, without other explanation than the SSc
b. Acute renal injury, without other explanation than the SSc AKI according to the KDIGO classification: more than 50% increase of serous creatinine from the reference value within the preceding 7 days or an absolute increase of 26.5 µmol/l (≥ 0.3 mg/dl) in 48 h
c. Thrombotic microangiopathy New or aggravated anemia without other explanation Schizocytes Thrombopenia ≤ 100,000/mm^3^ confirmed on a smear Signs of hemolysis: elevated LDH, low haptoglobin, increased reticulocytes Negative antiglobulin test
Target organ dysfunction Hypertensive retinopathy Hypertensive encephalopathy Pulmonary edema Acute pericarditis
Anomalies suggestive of renal biopsy (fibrinous thrombi, fibrinoid necrosis, glomerular collapse, onion bulb proliferation in pre-glomerular arterioles and arch arteries)

*HBP* high blood pressure, *BP* blood pressure, *SSc* systemic sclerosis, *AKI* acute kidney injury, *LDH* lactate dehydrogenase

9) with a sudden onset of severe hypertension accompanied by headache, visual disturbances, sometimes convulsions, heart failure, pericardial effusion, microangiopathic hemolytic anemia with schizocytes and thrombocytopenia with rapidly progressive oliguric renal failure. Microscopic hematuria, proteinuria with an extremely high plasma renin level is also sometimes found. This table may be incomplete.

**Table:**

Any de novo arterial hypertension and acute renal failure in a scleroderma patient should raise concerns of a scleroderma renal crisis.	

In the case of malignant arterial hypertension on renal attack, the initial severity is neurological (convulsions) and cardiopulmonary by the risk of acute pulmonary edema (APE).

Approximately 10% of scleroderma renal crisis are normotensive (Table

**Table 10:** Definition of normotensive scleroderma renal crisis

Normotensive scleroderma renal crisis
Creatinine levels over 50% above baseline or creatinine ≥ 120% of the upper normal range in the lab
**And one of the following five criteria:** At least one of the following 5 criteria: Proteinuria ≥ 2+ by strip Hematuria ≥ 2+ by strip or ≥ 10 RBC by field Thrombopenia < 100,000/mm^3^ Hemolysis defined by an anemia not linked to another cause with: (1) Schizocytes or other RBC fragments found on the blood smear (2) Increase in the reticulocyte level Renal biopsy showing a typical appearance of scleroderma renal crisis (fibrinous thrombi, fibrinoid necrosis, glomerular collapse, onion bulb proliferation in pre-glomerular arterioles and arch arteries)

10), which is difficult to diagnose.

Emergency diagnostic measures

- Formalized criteria for assessment of scleroderma renal crisis
- Assessing the severity:
- Malignant hypertension with neurological complications
- Threatening hyperkalemia on acute renal failure
- Hydro-sodium inflation requiring emergency extrarenal purification
- Severe respiratory distress requiring positive pressure, noninvasive or invasive ventilation
- Emergency exams:
- Urine test strip
- Electrocardiogram with at least 12 leads
- Blood count with schizocyte and platelet, creatinine, and kalemia counts

Immediate therapeutic measures (Fig. 5)

- Symptomatic measures:
- Treatment of hyperkalemia, extrarenal purification if necessary.
- Specific treatments:
- Refer to reference specialists or entrust to regional or national centers of reference for rare diseases (or centers of competence).

Emergency situation 2: Right cardiac decompensation on pulmonary arterial hypertension (PAH) Dyspnea is the main symptom. It often sets in gradually and insidiously. PAH can sometimes appear suddenly, for example during exertion (possible syncope of effort), during a high-altitude stay or when taking antiinflammatory drugs. It may be class IV dyspnea with signs of right heart decompensation. In the case of right heart decompensation on PAH, the clinical picture is obvious: lower limb edema, hepato-jugular reflux in a patient with known PAH. The risk is heart failure and syncope. If it is not inaugural, the evolution of such a clinical picture may be heightened with a shock on heart failure or consciousness disorders.

Emergency diagnostic measures

- Clinical elements of diagnosis:
- Clinical signs of right heart decompensation in a patient with known and treated PAH
- Right heart decompensation can sometimes be indicative of PAH
- Assessing the severity:
- Signs of respiratory distress, hypoxia, syncope, functional angina
- BNP or NT-proBNP tests
- Emergency exams:
- **If the diagnosis of PAH is not known,** the diagnosis is suspected on echocardiography, which finds dilatation of the right cavities and elevated pulmonary BP (PBP) with no sign of pulmonary embolism (post-embolic PH).

**Table:**

**Warning**	
In this context, the **angioscan** is used to evaluate thromboembolic disease (TED). It is imperative to use a **hypo-osmolar contrast agent with slow diffusion** because of the risk of defusing the cardiac pump in case of very high PBP.	

Integrated into an exhaustive check-up, not adapted to the context of the emergency department, right cardiac catheterization is necessary to confirm the diagnosis and direct the patient to the care channel best suited to his or her acuity subgroup.

**If PAH is known, an echocardiography** will assess immediate signs of severity. There is no indication to perform an emergency right heart catheterization.

Immediate therapeutic measures

- Symptomatic measures:
- Limit the patient’s efforts and any event likely to increase cardiac output (tolerance to the increase in afterload, but output limited by the preload, which cannot absorb the variations in venous return) or, conversely, related to hypovolemia, which may increase or cause coronary hypoperfusion.
- Oxygen therapy, diuretics depending on the clinic.
- Make sure that, if PAH is known and treated, that there have been no untimely treatment interruptions.
- An ultrasound of the vena cava will make it possible to evaluate the indication of diuretics at high doses (sometimes doses of up to 1.5 or 2 g/24 h of furosemide—40 mg of bumetanide are necessary).
- Preventive treatment with low molecular weight heparin to prevent TED, but the benefit of hypocoagulation is discussed in PAH associated with scleroderma and, on the other hand, there is a risk of gastrointestinal hemorrhage on telangiectasias.
- After management in the emergency department, treatment will require discussion of specific anti-PAH therapy adjustment: refer the patient to a specialized center such as a PAH reference or competence center.
- Specific treatments:
- Vasoactive amines if heart failure and low volume filling.
- Transfer to a reference or competence center for discussion of treatment by endothelin receptor antagonists, phosphodiesterase V inhibitors or prostacyclin (or in combination).
- **Discussion of a bipulmonary transplant in extreme emergency.**

Emergency situation 3: Acute decompensation of pulmonary fibrosis

The emergency is oxygenation by high-flow oxygen therapy or noninvasive or invasive mechanical ventilation.

Since PAH may be underlying in an unsuspecting and therefore untreated patient, there is a great deal of caution and therefore similarities in the emergency ventilatory approach with the emergency situation 2.

Emergency diagnostic measures

- Clinical elements of diagnosis:

Worsening dyspnea if pulmonary fibrosis known, cyanosis.

- Assessing the severity:

Altered consciousness, pause in breathing.

In case of abrupt respiratory aggravation in a scleroderma patient with known pulmonary fibrosis, look for:

- A bronchial or pulmonary superinfection
- A pulmonary embolism
- Left or global cardiac decompensation
- Onset or exacerbation of severe PH on fibrosis

If no embolism, no superinfection, and no evidence of PH, consider an acute exacerbation of pulmonary fibrosis.

Emergency exams:

As part of the recommendations for the evaluation of the etiologies mentioned in the previous paragraph, blood gas evaluation and chest CT imaging (more or less injected) are usual.

Immediate therapeutic measures

- Symptomatic measures:
- Oxygenation via a device adapted to the oxygen flow, possibly by noninvasive mechanical ventilation or even intubation and invasive ventilation.
- Respiratory physiotherapy in case of bronchial congestion.
- Antibiotic therapy in case of suspected infection.
- Specific treatments:
- Specific treatments should be discussed with specialists in a reference center or a competence center for rare diseases.
- No specific emergency treatment, symptomatic stabilization, and assessment of the depth of SSc signs.
- If acute exacerbation of pulmonary fibrosis with ARDS: consider high-dose corticosteroid therapy after discussion with a SSc reference or competence center.

Emergency situation 4: gastrointestinal bleeding

It can occur on esophageal ulcers or on gastric, duodenal or even small intestine or colon telangiectasias.

Emergency diagnostic measures

- Clinical elements of diagnosis:

Anemia on digestive bleeding.

- Assessing the severity:
- Hemodynamic status
- Severity criterion: “watermelon” stomach evaluated by gastric fibroscopy
- Emergency exams:
- CBC-platelets, hematocrit, pretransfusion workup
- Emergency endoscopy: confirms the diagnosis and allows treatment
- Symptomatic (laser if telangiectasias)

Immediate therapeutic measures

- Symptomatic measures:
- Blood transfusion if necessary
- Admission to the ICU
- Intravenous iron
- Specific treatments:
- Hemostatic laser treatment
- Antral surgical resection (if severe bleeding in the “watermelon” stomach)

Emergency situation 5: Decompensation of scleroderma cardiomyopathy

Pericardial, myocardial or, more rarely, endocardial damage.

Most often secondary to PAH or PH secondary to pulmonary fibrosis causing progressive dilation of the right cavities, sometimes resulting in right heart failure.

Sometimes primitive, specific to SSc, often not very symptomatic.

Usually, there is no damage to the coronary trunks, but the coronary reserve is defective with a functional inability of the microcirculation to adapt to the increased cardiac work, causing myocardial ischemia with a risk of conduction or rhythm disturbance.

Emergency diagnostic measures

- Clinical elements of diagnosis:

The clinical expression is variable:

- Absence of symptoms or chest pain, healthy coronary arteries, rhythm or conduction disturbances
- When myocardial fibrosis affects the left cavities: frequent alteration of ventricular diastolic function with systolic function maintained for a long time
- Brain natriuretic peptide test: BNP/NT-proBNP
- Assessing the severity:
- Type IV dyspnea of the functional classification of heart disease according to the New York Heart Association (NYHA)
- Cyanosis
- Right or left heart failure
- Look for a ventricular rhythm disorder and a conduction disorder (risk of sudden death)
- Emergency exams:
- EKG
- Echocardiogram
- BNP/NT-proBNP test
- Troponin

Coronary angiography according to the clinical presentation, biological and electrical data (most often normal, the cardiac involvement of scleroderma is often microcirculatory).

**Immediate therapeutic measures**

- Monitoring:

Continuous EKG: elevated risk of heart rhythm disorders. Continuous electrocardiographic monitoring in the cardiac intensive care unit or intensive care unit.

- Symptomatic measures:

Usual measures: low-salt diet, ACE inhibitors II, diuretics

Calcium channel blockers of the dihydropyridine family can improve myocardial perfusion

- Specific treatments:

Refer the patient to specialists in reference or competence centers for an assessment of all potential SMA impairments.

Emergency situation 6: digestive motility disorder

The clinical expression depends on the primary level of impairment:

- Esophagus (75–90% of patients): pyrosis, dysphagia, peptic esophagitis
- Stomach (50% of patients): early feeling of fullness, nausea, vomiting, bloating
- Small intestine (40–70% of patients): diarrhea, constipation, digestive distension, malabsorption syndrome, microbial swelling, pseudo-obstructive syndrome
- Colon (20–50% of patients): colonic distension, constipation, pseudobstructive syndrome

1.** Emergency diagnostic measures**

- Clinical elements of diagnosis:

Sub-occlusive or occlusive state.

- Assessing the severity:

Complications: fecal impaction, perforations, volvulus, cholestasis, digestive infarction.

- Emergency exams:

The plain abdominal X-ray can be used to look for signs of occlusion, sternal stasis, but the abdominal CT scan is much more efficient.

2. **Immediate therapeutic measures**

- Monitoring:

Monitoring of intestinal transit, beware of false diarrhea on chronic constipation, make sure there is no obstructive fecal impaction.

- Symptomatic measures:

**Table:**

**Warning:** Remove an obstruction on fecal impaction.	

- If pseudo-obstructive syndrome or obstipation with digestive distension: octreotide (recommended initial dose of 50 µg × 2/day, to be increased if clinical response is inadequate, but not to exceed 100 µg × 2/day) may be effective.
- If colic attack: hospitalization (sometimes prolonged), manual evacuation if obstructive fecal impaction occurs, use of laxatives and enemas in combination with a high-fiber diet.
- Serious complications can occur such as stercoral perforation, volvulus, cholestasis and even digestive infarction.
- Prokinetic drugs are not very effective.

Specific treatments:

Refer the patient to specialists in reference or competence centers with expertise on rare autoimmune diseases for assessment of all visceral disorders.

**B. Drug precautions (possible interactions, contraindications, precautions for use, etc.)**

Never abruptly stop PAH treatments (sildenafil, tadalafil, bosentan, ambrisentan, selexipag, intravenous epoprostenol, subcutaneous treprostinil, aerosolized iloprost): risk of cardiac decompensation.

In patients treated with a phosphodiesterase V inhibitor (sildenafil, tadalafil) for PAH: major risk of vasoplegic shock if the patient is treated with nitrates or analogues.

**C. Technical precautions and prevention of complications**

- Systemic damage, particularly to the lungs or heart, can limit the indications for general anesthesia (GA) and requires great vigilance in the choice of the mode and parameters of invasive or noninvasive ventilation.
- GA indications must be discussed and put into perspective with any anticipated directives.
- For the performance of certain procedures indicating a GA, give preference to alternative techniques (locoregional anesthesia, anesthesia with a concentration objective).
- Venous vascular edges: peripheral perfusion is often difficult, with frequent use of central venous lines.
- Arterial vascular edges: significant risk of digital ischemia or thenar eminence when inserting radial catheters: perform an Allen’s test and discuss a first femoral approach despite a higher risk of infection.
- Vasopressor amines are associated with a high risk of digitalis vasoconstriction and digital necrosis.
- Potentially difficult endotracheal intubation (microstomia, cervical stiffness). Anticipate a difficult intubation procedure with experienced operators: smaller probe diameter, Eichmann type clamp, video laryngoscopy, fibroscopy.
- Special monitoring of the patient in the immediate aftermath of an extubation (high risk of aspiration due to gastroesophageal reflux).
- In resuscitation, in an unintubated patient, especially when resuming oral feeding:
  - Prevent gastroesophageal reflux, as there is a major risk of inhalation pneumopathy.
  - If the patient is suffering from severe reflux, maintain a 45° semi-seated Trendelenburg position.
  - Nutritional support by preferential enteral route. The insertion of the gastric tube may be made difficult by esophageal motility disorders and must sometimes be inserted during a gastric fibroscopy.
  - Passive physiotherapy in sedated patients, then active as soon as possible to prevent joint retraction and amyotrophy.

**D. Organ and tissue donation**

In the current state of knowledge, the donation of certain organs and tissues may be possible pending a case-by-case analysis.

1.** Risk of disease transmission**

In the absence of evidence in the literature, since the exact cause of the disease is related to an autoimmune reaction for which the triggering factors are not known, it is not possible to make a statement on this risk.

Corticosteroids and long-term immunosuppressants (cyclophosphamide, methotrexate, mycophenolate mofetil, azathioprine, etc.) cause a decrease in the donor’s immune response to certain transmissible infectious diseases (acute or not), with a risk of delayed or weak serological reactions that are difficult to diagnose. The use of viral genomic screening is the solution.

2.** Organ donation: depending on the type**

- Limited cutaneous SSc: no contraindication for liver (in the absence of associated primary biliary cirrhosis) and pancreas donation. For other organs, a careful assessment is required.
- Diffuse cutaneous SSc: the validation for organ donation needs to be more cautious; liver and pancreatic sampling may be considered. Above all, do not underestimate the possibility of kidney damage, as kidney sampling is then impossible.

Some studies show that, even when asymptomatic, 69% of hearts and 50% of kidneys (pre-implantation biopsy is often recommended) are affected by the disease. The transplant decision is therefore based on the team’s estimate of the risk incurred by the recipient in relation to the expected benefits of the transplant (benefits & risks).

3.** Tissue donation**:

The European guide contraindicates SSc for bone and skin sampling. It also seems reasonable to contraindicate donations of vascular tissue and heart valves. The inherent risks in long-term treatment followed by the donor must be known and taken into account.

Corneal collection is possible except when there is an association with severe Sjögren’s syndrome with corneal damage. It then seems reasonable to contraindicate the collection unless prior ophthalmological advice is given.

ABM’s Regulatory and Field Support Services (SRA): numbers for the four regulatory territories (24/7)

**Table:**

SRA Northeast	+ 33	9	69	32	50	20
SRA Southeast/Indian Ocean	+ 33	9	69	32	50	20
SRA Great West	+ 33	9	69	32	50	20
SRA Ile de France/Center/West Indies/French Guiana	+ 33	9	69	32	50	20

## Appendix 8: List of useful links for healthcare professionals and patients

Information for healthcare professionals:

- FAI^2^R—Health Network for Rare Autoimmune and Autoinflammatory Diseases, www.fai2r.org
- GFRS—Francophone Scleroderma Research Group, www.sclerodermie.net
- EUSTAR—EULAR scleroderma trials, www.eustar.org
- Orphanet, www.orpha.net/
- SNFMI—French National Society of Internal Medicine, www.snfmi.org
- SFR—French Rheumatology Society, http://sfr.larhumatologie.fr
- SFD—French Dermatology Society, http://www.sfdermato.org
- SFMC—French Microcirculation Society
- SFMV—French Society of Vascular Medicine, http://www.portailvasculaire.fr/espace-sfmv/accueil
- SFGM-TC—Francophone Society of Bone Marrow Transplantation and Cellular Therapy, http://www.sfgm-tc.com
- SOFREMIP—Francophone Society for Rheumatology and Pediatric Inflammatory Diseases, www.sofremip.sfpediatrie.com
- Reference Center for Rare Pulmonary Diseases, www.maladies-pulmonaires-rares.fr

Information for patients:

- ASF—French Scleroderma Patient Association, www.association-sclerodermie.fr
- Rare Disease Alliance, www.alliance-maladies-rares.org
- EURORDIS—Federation of Associations of Patients and Individuals Active in the Domain of Rare Diseases, www.eurordis.org
- FAI^2^R—Health Network for Rare Autoimmune and Autoinflammatory Diseases, www.fai2r.org
- FMO—Orphan Disease Federation, www.maladies-orphelines.fr
- Rare Disease Info Services—www.maladiesraresinfo.org

## Abbreviations

ANA: Antinuclear antibodies
Ab: Antibody
ACR: American College of Rheumatology
GA: General anesthesia
NSAID: Nonsteroidal antiinflammatory drug
ALD: *Affection de longue durée* [long-term illness], also refers to part of the French national healthcare system
MA: Marketing authorization
ANCA: Anti-neutrophil cytoplasmic antibody
ARB: Angiotensin II receptor blockers
ARS: *Agence régionale de santé* [regional health agency]
ALT: Alanine transaminase
AST: Aspartate transaminase
BAFF: B-cell activating factor
BNP: B-type natriuretic peptide
CCP: Cyclic citrullinated peptide
CPK: Creatine phosphokinase
TLC: Total lung capacity
CRP: C-reactive protein
HSC: Hematopoietic stem cells
FVC: Forced vital capacity
DLCO: Carbon monoxide diffusing capacity
cDLCO: Corrected carbon monoxide diffusing capacity
DMARD: Disease-modifying antirheumatic drug
ECG: Electrocardiogram
PFT: Pulmonary function tests
ELISA: Enzyme-linked immunosorbent assay
TPE: Therapeutic patient education
EULAR: European League Against Rheumatism
EUSTAR: European Scleroderma Trials and Research Group
SA: Spontaneous abortion
LVEF: Left ventricular ejection fraction
FGF: Fibroblast growth factor
FT4: Free thyroxine
γGT: Gamma-glutamyltransferase
HAS: High Health Authority [of France]
HBP: High blood pressure
PAH: Pulmonary arterial hypertension
PH: Pulmonary hypertension
ACEI: Angiotensin-converting enzyme inhibitor
IL: Interleukin
BMI: Body mass index
PPI: Proton pump inhibitor
MRI: Magnetic resonance imaging
BAL: Bronchoalveolar lavage
LDH: Lactate dehydrogenase
MCP: Metacarpophalangeal
MMF: Mycophenolate mofetil
mRSS: Modified Rodnan skin score
MTX: Methotrexate
CBC: Complete blood count
NT-proBNP BNP: N-terminal pro b-type natriuretic peptide B-type natriuretic peptide
NYHA: New York Heart Association
WHO: World Health Organization
PAP: Pulmonary arterial pressure
ILD: Interstitial lung disease
NDCP: National diagnostic and care protocol
IUGR: Intrauterine growth restriction
MCM: Multidisciplinary coordination meeting
GERD: Gastroesophageal reflux disease
PVR: Pulmonary vascular resistance
WOA: Week of amenorrhea
SSc: Systemic scleroderma
SLS: Scleroderma Lung Study
aPTT: Activated partial thromboplastin time
PT: Prothrombin time
TSH: Thyroid-stimulating hormone
VEGF: Vascular endothelial growth factor
LV: Left ventricle
HIV: Human immunodeficiency virus
TRV: Tricuspid regurgitation velocity

## Footnotes

1 As an exception, in particular when the diagnosis is made in the hospital or on an emergent basis, another doctor may establish the care protocol. Then, 100% coverage can be provided for a period of 6 months, with possible renewals.
